# A 3D spheroid model for assessing nanocarrier-based drug delivery to solid tumors

**DOI:** 10.1038/s44385-025-00041-x

**Published:** 2025-10-24

**Authors:** Chitra Yadav, Alexander S. Evtushenko, Andrea Bistrović Popov, Beatriz Lozano Torres, Ishtiaq Ahmed, Liuba Dvinskikh, Clemens F. Kaminski, Ljiljana Fruk

**Affiliations:** Department of Chemical Engineering and Biotechnology, University of Cambridge, Philippa Fawcett Drive, Cambridge, UK

**Keywords:** Nanoparticles, Cancer models, Pancreatic cancer

## Abstract

3D spheroid culture has emerged as a valuable tool for studying complex intratumoral processes and screening novel therapeutics in vitro. However, spheroids face reproducibility and data interpretation issues, which limit their utility. This work describes a simple and reproducible co-culture spheroid model compatible with high-throughput screening designed to study pancreatic ductal adenocarcinoma (PDAC), a highly therapy-resistant cancer. These spheroids, composed of both cancer and stromal cells, recapitulate key features of PDAC which are difficult to study in traditional 2D cell culture, including hypoxia, fibrosis and chemoresistance. Light sheet microscopy is used to study the tissue penetration of polymeric Pluronic® F127-polydopamine (PluPDA) nanocarriers (NCs) in this model while showing that confocal microscopy is not suitable for such studies and should be avoided. Additionally, the efficacy of PluPDA NCs loaded with the chemotherapeutic SN-38 is demonstrated in 3D, justifying their advancement to in vivo trials. Finally, the methodology is extended to generate lung adenocarcinoma spheroids, showcasing the versatility of this approach. Overall, this research is intended to serve as a robust platform for studying NCs under physiologically relevant conditions, ultimately resulting in a more efficient clinical translation pathway for nanomaterials.

## Introduction

Over 90% of anti-cancer clinical trials fail^1^, predominantly due to the drugs investigated lacking clinical efficacy or showing unmanageable toxicity^2^. A key reason for this high failure rate is the inaccuracy of currently available pre-clinical cancer models, both in vitro and in vivo, which has recently been recognized as one of the principal challenges facing cancer research^3^. Early-stage pre-clinical studies continue to rely heavily on two-dimensional (2D) in vitro cell culture, whereby cells are grown as monolayers on rigid adhesive surfaces. This approach remains widely used due to its relative simplicity, low cost and compatibility with high-throughput screening^4^. However, as our understanding of cancer biology advances, it is becoming increasingly clear that 2D culture fails to replicate the complex three-dimensional architecture and cellular interactions of solid tumors, limiting its utility as a physiologically accurate disease model^5^. In vivo, the tumor microenvironment (TME) is composed of cancer, stromal, and immune cells, along with vasculature and extracellular matrix (ECM)^6,7^, all of which engage in continuous crosstalk. Additionally, real tumors develop chemical gradients, resulting in spatially heterogeneous oxygenation^8^, pH^9^, mechanical stiffness^10^ and drug penetration^11^. These factors significantly impact a tumor’s response to any treatment, yet none of them can be adequately replicated using conventional 2D cell culture.

**Fig. 1:**
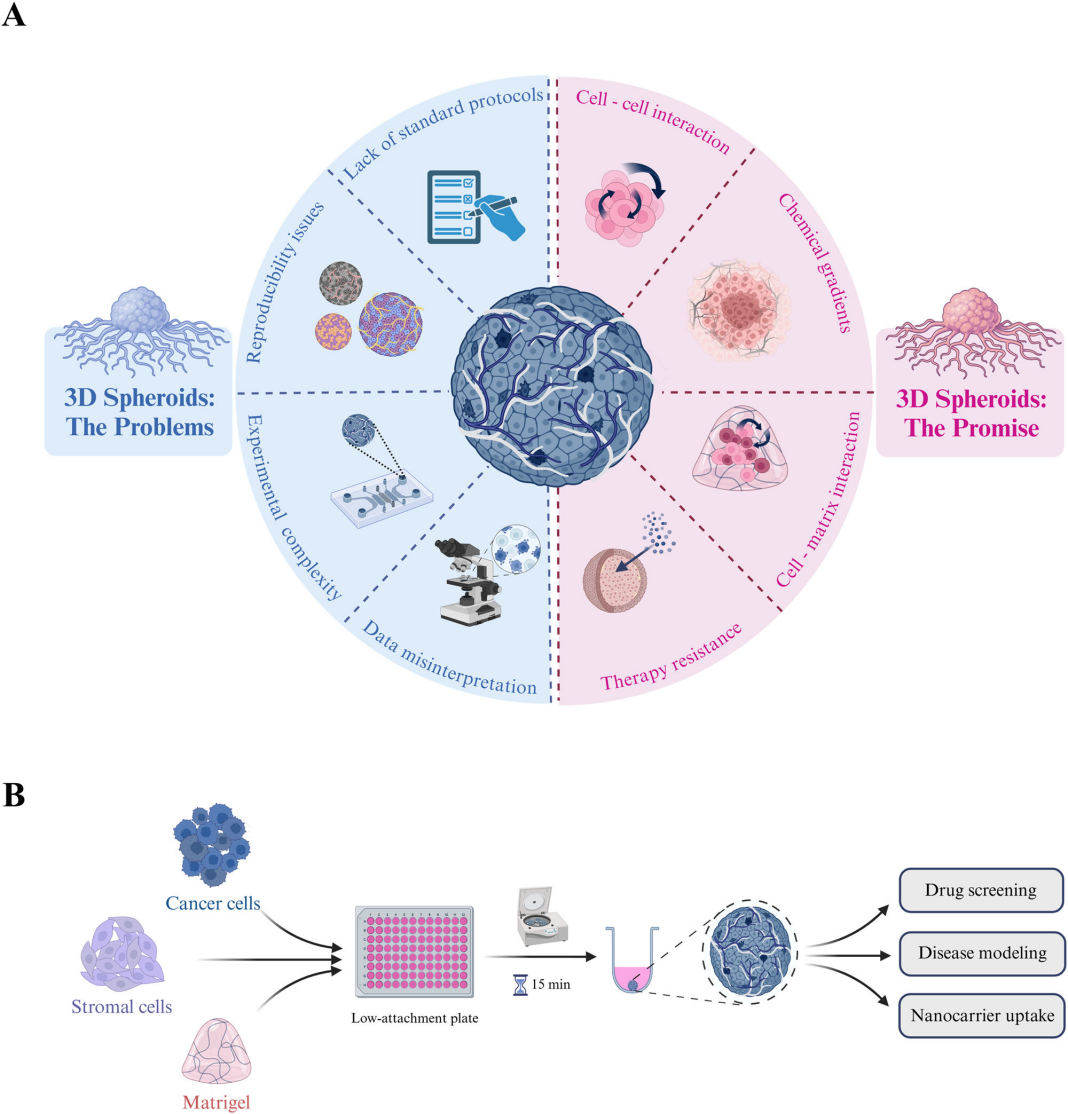
An introduction to 3D spheroid culture. **A** Graphical summary of the benefits and challenges of 3D spheroid culture. **B** Schematic of the experimental pipeline for spheroid generation used in this study (created using Biorender.com).

Given these limitations, three-dimensional (3D) cell culture has emerged as a powerful tool for modeling complex intra-tumoral interactions in vitro^12^. Among 3D models, tumor spheroids offer a unique combination of simplicity, reproducibility and physiological relevance (Fig. 1A). Spheroids form when cells are cultured without an adhesive surface, promoting cell-cell attachment and the development of structures representative of real tumor organization^13^. Unlike 2D, spheroid cultures can incorporate ECM components and stromal cells, allowing for the modeling of complex TME dynamics^14^. This is particularly relevant in highly desmoplastic tumors such as pancreatic ductal adenocarcinoma (PDAC), where the TME plays a critical role in therapy resistance^15^. Indeed, it has been shown that PDAC cells cultured as spheroids are significantly less susceptible to chemotherapy than those grown in 2D^16^, mirroring the high degree of chemoresistance observed in vivo^17^. Additionally, the gene expression profiles of cells in spheroids have been shown to more closely match those of real tumors compared to cells grown in 2D^18^. By providing a physiologically accurate setting for studying cancer therapy response, spheroids can facilitate the early identification of ineffective drug candidates, preventing their advancement to costly and time- and resource-intensive in vivo trials. Furthermore, patient-derived spheroids are increasingly being employed in clinical precision medicine, with several clinical trials demonstrating the successful use of biopsy-derived cells to generate spheroids for drug screening and the selection of optimal patient-specific treatments^19,20^.

Consequently, one research area that could greatly benefit from a more widespread adoption of spheroid culture is cancer nanomedicine, which focuses on the development of nano-structured therapeutic agents. Over the past 30 years, sixteen nanocarrier (NC)-based therapeutics have received regulatory approval for cancer treatment^21,22^, notably including liposomal irinotecan (Onivyde®) and albumin-bound paclitaxel (Abraxane®), both of which are used in PDAC management^23^. NCs offer several clinical benefits by protecting molecular drugs from degradation, improving pharmacokinetics, enhancing tumor accumulation, circumventing drug resistance, and minimizing systemic side effects^24,25^. Recently, the success of NC-based mRNA vaccines during the COVID-19 pandemic has further fueled interest in nanomedicine, with the global nanotechnology market projected to double in size from $7.1 billion in 2020 to $13.6 billion by 2027^26,27^. In spite of this, the majority of promising novel nanotherapeutics fail to reach the clinic^28^, highlighting the urgent need for more predictive pre-clinical models.

Although spheroids are well-suited to addressing this issue, their widespread adoption in nanomedicine research is hindered by several factors. Firstly, the great variety of spheroid generation techniques developed to date^29,30^ makes it difficult to directly compare results across studies, reducing data reliability compared to standardized 2D cultures. The historically popular hanging drop method, in which cells are seeded into small droplets of medium suspended from a horizontal surface, has increasingly come under criticism, as the resulting spheroids are difficult to handle, dose with drugs, and image, and typically require transfer to conventional microplates for most downstream applications^31,32^. Conversely, several more recently developed spheroid models rely on highly specialized equipment such as microfluidic systems or custom-made scaffolds, which can limit reproducibility and accessibility^33^. Furthermore, in our experience, spheroid data in nanomedicine papers often suffer from incomplete and occasionally incorrect analysis, limiting their utility.

To address these issues, we hereby present a simple and reproducible protocol for the generation of PDAC spheroids (Fig. 1B) as well as the analytical methods used to assess their morphology, growth dynamics, and treatment response. Notably, our model accurately recapitulates PDAC characteristics such as hypoxia and fibrosis which are difficult to study in 2D and can be used to assess the efficacy of small-molecule drugs and drug-loaded NCs. Additionally, we identify common methodological pitfalls that hinder the utility of spheroid experiments and provide practical solutions to mitigate them. Finally, we demonstrate that our protocols can be readily adapted to lung adenocarcinoma, showcasing their versatility across different malignancies. Ultimately, we aim to establish a reproducible, cost-effective, and physiologically accurate platform for the in vitro evaluation of drugs and NCs, paving the way to a more efficient pathway for clinical translation.

## Results

### Morphology and growth dynamics of PDAC spheroids

PDAC is a genetically heterogeneous cancer, which cannot be accurately represented by a single in vitro model^34^. To address this, we developed two distinct models using different PDAC cell lines: PANC-1, a *KRAS*^*G12D*^ mutant that represents the most common PDAC driver mutation; and BxPC-3, a wild-type *KRAS* cell line representing a clinically significant subset of PDAC with better prognosis^35^. Additionally, we co-cultured both cell lines with pancreatic stellate cells (hPSCs), a major source of PDAC cancer-associated fibroblasts (CAFs)^36^. CAFs are a heterogeneous population of non-cancerous stromal cells involved in ECM synthesis and pro-tumorigenic cytokine secretion, hence hPSCs were incorporated in the models to more accurately represent the complexity of the PDAC TME^37^.

**Fig. 2:**
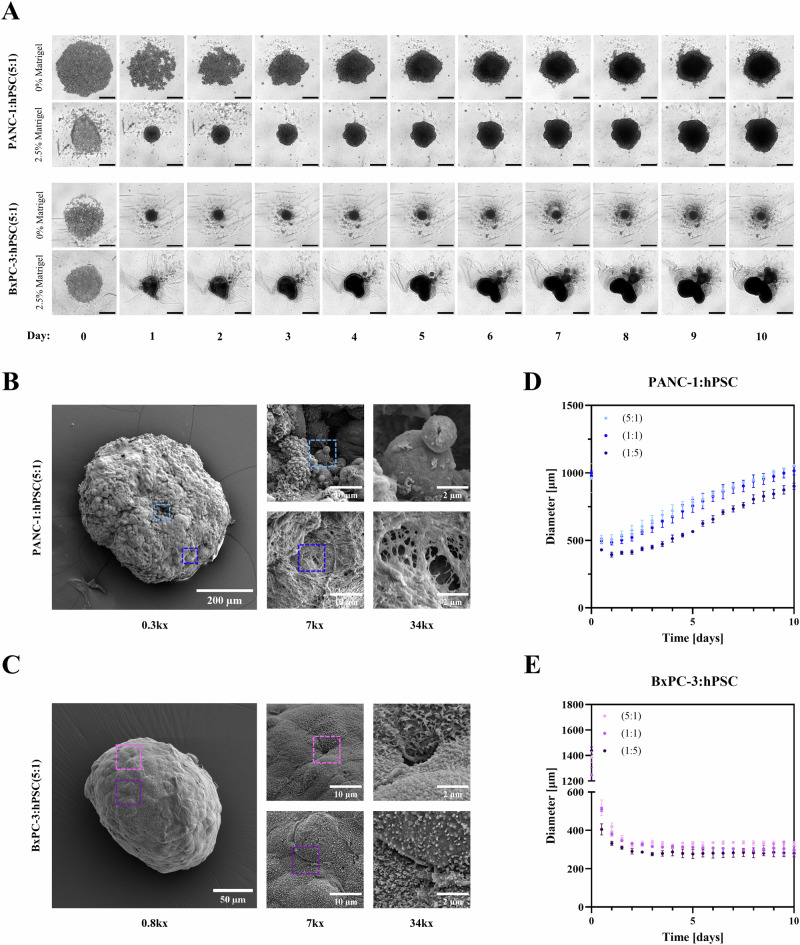
Characterization of spheroid size, morphology and growth dynamics. **A** Incucyte® images of PANC-1:hPSC(5:1) and BxPC-3:hPSC(5:1) spheroids grown with and without supplementation with 2.5% Matrigel® for 10 days. Scale bars represent 500 µm. **B** Scanning electron microscopy characterization of PANC-1:hPSC(5:1) and **C** BxPC-3:hPSC(5:1) spheroids showing different surface morphologies. **D** Evolution of PANC-1:hPSC and (**E**) BxPC-3:hPSC spheroid diameter over time, calculated from Incucyte® images, as a function of cancer-to-stellate cell ratio.

We generated spheroids by mixing PDAC cells and hPSCs in low-attachment 96-well plates, centrifuging them to force the cells close together and promote cell-cell contact, then incubating them under standard tissue culture conditions. Spheroid formation and subsequent growth were monitored using an Incucyte® live-cell analysis system. Notably, the two PDAC cell lines resulted in markedly different spheroids when grown under the same conditions. Initially, PANC-1:hPSC spheroids were large, loosely packed and easily dissociated into individual cells, making them difficult to handle (Fig. 2A, Supplementary Video 1). Such structures should not be referred to as “spheroids” despite their approximately spherical nature, since loose cell aggregates are unlikely to accurately model the dense architecture of a solid tumor such as PDAC. To remedy this, we supplemented PANC-1:hPSC culture medium with 2.5% Matrigel®, resulting in much smaller and denser spheroids which steadily grew from ~500 µm to ~1 mm in diameter by day 10 (Fig. 2A, D, Supplementary Video 2). At lower Matrigel® concentrations (0–1.25%), the spheroids remained loosely packed, suggesting 2.5% as the minimum effective concentration (Fig. S1).

Conversely, BxPC-3:hPSC spheroids were dense and relatively small (~300 µm in diameter) even in the absence of Matrigel®. These spheroids took 2 days to fully form, after which their size remained constant (Fig. 2A, E, Supplementary Video 3). From day 5 onwards, clearly visible debris were observed, suggesting cell death and restricting these spheroids’ use in NC studies to days 2–5. Unlike PANC-1:hPSC, the addition of 2.5% Matrigel® to the medium of BxPC-3:hPSC resulted in large irregularly shaped 3D structures with significant morphological variation between replicates (Fig. 2A, Supplementary Video 4). To ensure reproducibility, we therefore used Matrigel®-free medium for this model.

Matrigel®, composed of ~60% laminin and ~30% collagen IV^38^, was selected for our model owing to its widespread use in PDAC organoid culture^39^. However, while laminin is a component of the PDAC ECM, the ECM in patient tumors is, on average, composed of over 90% collagen^40^, a feature that Matrigel® does not accurately replicate. To address this, we also generated spheroids in media supplemented with 15–60 µg/mL collagen I and monitored their size and morphology over time (Fig. S2A, C). Similarly to Matrigel®, collagen I supplementation increased the uniformity and compaction of PANC-1:hPSC spheroids, while producing highly irregular and heterogenous BxPC-3:hPSC spheroids. Consistently with previous reports^41^, collagen I also induced marked invasiveness in PANC-1:hPSC spheroids, with the extent of invasion increasing in a collagen concentration-dependent manner (Fig. S2B). For subsequent experiments, Matrigel® was used for PANC-1:hPSC spheroids to ensure all observations were specific to purely 3D cultures with no 2D cell outgrowth. Nevertheless, collagen-supplemented spheroids may serve as a valuable model for studying invasion and metastasis in future applications.

Both spheroid types appeared uniform under light microscopy, with PANC-1:hPSC and BxPC-3:hPSC exhibiting over 75% and 85% circularity respectively and having aspect ratios under 1.2 (Fig. S3). To better examine their morphology, the spheroids were fixed, dehydrated and observed using scanning electron microscopy (SEM). PANC-1:hPSC spheroids displayed heterogeneous surfaces with alternating regions of exposed cells and ECM fibers (Fig. 2B). Conversely, BxPC-3:hPSC spheroids had smooth homogeneous surfaces comprised of tightly packed cells with no visible ECM deposits (Fig. 2C). These stark morphological differences underscore the need for using multiple cell lines to model PDAC in vitro.

We finally investigated the effects of varying the ratio of PDAC cells to hPSCs on spheroid growth. While it is often stated that the PDAC stroma comprises up to 90% of tumor volume^42,43^, this does not mean that 90% of the cells in a PDAC tumor are stromal cells. In fact, the ratio of cancer to stromal cells varies greatly between patient samples^37^, ranging from 95% to 14% cancer cells^44^ and making it difficult to establish a universally accurate seeding ratio for in vitro models. Consequently, we prepared spheroids using 5:1, 1:1 and 1:5 ratios of PDAC cells to hPSCs while maintaining a constant total number of cells per spheroid. In PANC-1:hPSC, the 5:1 and 1:1 ratios exhibited similar growth dynamics, while 1:5 spheroids were consistently 10–20% smaller, suggesting that hPSCs proliferate more slowly than PANC-1 in 3D (Fig. 2D). Conversely, BxPC-3:hPSC spheroid diameter was independent of cell ratio (Fig. 2E). Since these spheroids did not extensively proliferate after formation, the presence of more slowly dividing hPSCs did not affect their size. Altogether, these findings showcase the adaptability of our model to different clinical sub-types of PDAC, highlighting its potential for studying tumor heterogeneity in vitro.

### Internal organization of PDAC spheroids

**Fig. 3:**
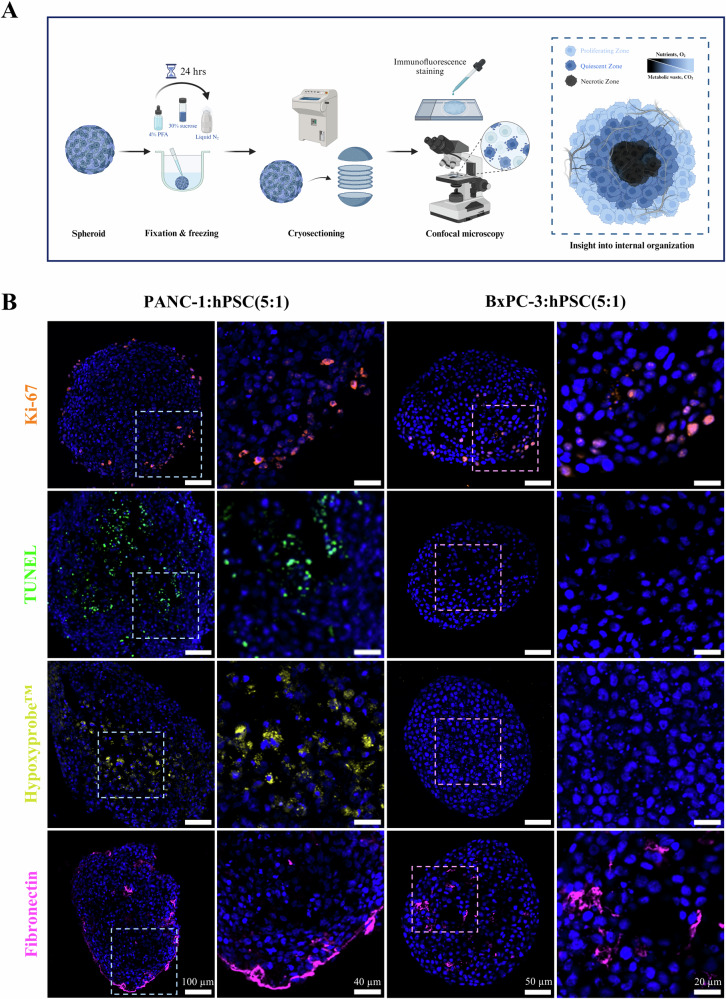
Characterization of internal spheroid organization by confocal microscopy. **A** Schematic representation of the experimental pipeline used for spheroid sectioning, staining and imaging (created with Biorender.com). **B** Confocal microscopy images showing PANC-1:hPSC(5:1) and BxPC-3:hPSC(5:1) spheroid sections stained for proliferation (Ki-67 immunostaining, orange), apoptosis (TUNEL assay, green), hypoxia (Hypoxyprobe™ assay, yellow) and ECM deposition (fibronectin immunostaining, pink).

PDAC tumors exhibit spatial heterogeneity^45^ characterized by desmoplasia^46^ and the development of nutrient-poor and hypoxic regions^47^, none of which can be adequately replicated using 2D culture. To determine whether such heterogeneity could be reproduced in 3D, spheroids were fixed, sectioned, stained for a range of biomarkers and imaged using confocal microscopy (Fig. 3A). We analyzed four key biological processes, namely cell proliferation using Ki-67 immunostaining, apoptosis using a TUNEL assay, hypoxia using a Hypoxyprobe™ kit, and ECM deposition using fibronectin (FN) immunostaining (Fig. 3B). For illustration, fluorescence intensity profiles were generated along a line drawn across each spheroid section (Fig. S4A, B). Since such profiles are inherently biased, we also performed more rigorous quantification by integrating the intensity of each biomarker signal across each section’s entire area, then plotting it as a function of distance from spheroid center (Fig. S4C, D).

In both models, Ki-67 expression was observed predominantly in the outer 20% of spheroid radius, with minimal positive staining in the core. In PANC-1:hPSC(5:1), both TUNEL and Hypoxyprobe™ staining was conversely confined to the spheroid core, with little fluorescent signal observed in the proliferative outer layer. This pattern is consistent with the observation that chemical gradients develop within spheroids^18^. The proliferative outer edge is exposed to a high nutrient concentration and approximates blood vessel-adjacent tumor regions, while the spheroid core becomes nutrient-poor and hypoxic, leading to cellular quiescence and ultimately death. In contrast, BxPC-3:hPSC(5:1) spheroids exhibited no detectable apoptosis nor hypoxia. It has been previously reported that the establishment of nutrient gradients and the subsequent development of apoptotic cores require a spheroid diameter of at least 500 µm^48^, which was not attained by this model. Nevertheless, BxPC-3:hPSC spheroids can serve as a hypoxia-negative system representing well-vascularized tumors, complementing the hypoxic PANC-1:hPSC model.

Finally, we visualized ECM deposition by staining FN, a notable component of PDAC ECM with a key role in pro-tumorigenic signaling^49^. In PANC-1:hPSC spheroids, FN was mainly observed as a thin outer layer, likely corresponding to the fibrous ECM structures observed by SEM (Fig. 2B). Notably, since FN is not a significant component of Matrigel®^38^, this coating was likely synthesized by the spheroids themselves. Furthermore, FN-rich regions were also detected in Matrigel®-free BxPC-3:hPSC, confirming that the spheroids actively produce their own FN. However, unlike PANC-1:hPSC, the FN deposits in BxPC-3:hPSC were predominantly internal, with negligible spheroid surface expression, again agreeing with SEM imaging (Fig. 2C).

Both spheroid models faithfully reproduced several key hallmarks of PDAC tumors that are difficult to replicate in 2D culture. Given that both FN^50^ and hypoxia^51^ play well-established roles in PDAC therapy resistance, these models provide a valuable platform for testing new treatments targeting stromal remodeling and hypoxia relief. Rigorous analysis of spheroid section images before and after such treatments can yield a quantitative measure of treatment efficacy, enabling more predictive pre-clinical studies.

### Nanocarrier uptake in PDAC spheroids

A key advantage of spheroids over 2D cell culture is their ability to model the tissue penetration of drugs and NCs. In 2D monolayer cultures, all cells are exposed to the same NC concentration, leading to uniform internalization rates. Conversely, NC penetration in spheroids depends on diffusion through the ECM and/or transcytosis, mimicking some of the biological barriers present in real tumors.

**Fig. 4:**
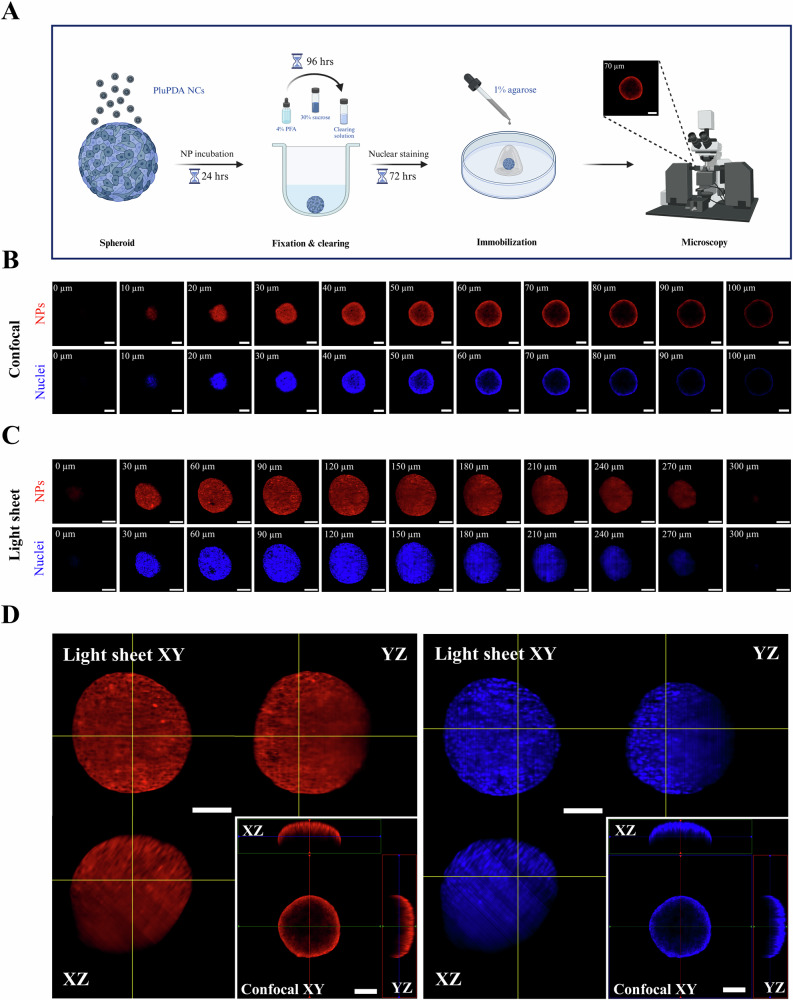
Microscopy techniques to study NC penetration into spheroids. **A** Schematic representation of the experimental pipeline used for spheroid preparation and imaging (created with Biorender.com). **B** Z-stack confocal laser scanning microscopy images of a BxPC-3:hPSC spheroid incubated with rhodamine B-labeled polydopamine-Pluronic® F127 NCs. Optical sections are shown for every 10 µm up to a depth of 100 µm. **C** Light sheet fluorescence microscopy images of the same spheroid. Optical sections are shown for every 30 µm up to a depth of 300 µm. **D** Orthogonal slices of the whole spheroid generated for both techniques. In all images, NCs are shown in red and cell nuclei, stained with SYTOX™ Green, are shown in blue. All scale bars represent 100 µm.

To explore NC uptake in spheroids, we used biocompatible polydopamine-Pluronic® F127 (PluPDA) NCs previously developed and characterized by us and used for drug delivery to PDAC cells in 2D^52^. Spheroids were treated with 50 µg/mL of drug-free 100 nm NCs labeled with rhodamine B (Fig. S5A–C) for 24 hours, then fixed, optically cleared using the Sca*l*eA2 method^53^, immobilized in agarose gel, and finally imaged using two different fluorescent microscopy techniques (Fig. 4A): confocal laser scanning microscopy (CLSM) and light sheet fluorescence microscopy (LSFM). CLSM employs pinhole apertures to reject out-of-focus light, enabling optical sectioning and improving lateral resolution. CLSM is widely used in life sciences research with many commercially available instruments^54^. On the other hand, LSFM illuminates samples with a thin sheet of light to excite fluorescence in a single optical section, allowing for fast volumetric imaging up to a depth of several hundred microns in scattering tissue and several millimeters after optical clearing^55–57^. Though less common than CLSM, LSFM has been successfully used to study the uptake of polymeric^58^, carbon-based^59^, lipid-based^60^ and silica-based^61^ NCs in spheroids.

Initially, CLSM indicated limited NC penetration in both spheroid models, with strong fluorescence observed in the spheroid periphery but little signal beyond a depth of 100 µm (Fig. 4B, D, S6, Supplementary Videos 5–8). Quantitative CLSM image analysis suggested that PluPDA NCs penetrated the outer 73.8% of BxPC-3:hPSC and 41.7% of PANC-1:hPSC spheroid radii, corresponding to relative volumetric penetrations of 98.2% and 80.2% respectively (Fig. S7–9). However, LSFM produced directly opposite results, revealing homogeneous rhodamine signals throughout both models (Fig. 4C, D, S6–9, Supplementary Videos 9–12) and suggesting that 24 hours was sufficient for the NCs to fully penetrate the whole spheroids. While these findings showcase the high tissue penetration of PluPDA NCs, they also highlight key differences between the two imaging techniques.

CLSM suffers from a fundamentally limited tissue penetration due to its rejection of out-of-focus scattered light. While this feature gives it excellent lateral resolution, it also restricts its penetration depth to less than 100 µm^58,62^, as clearly illustrated by Fig. 4B. As such, CLSM is fundamentally unsuitable for studying the penetration of any material in whole 3D tissues, and its use should be limited to thin tissue sections. Unfortunately, numerous published nanomedicine studies appear to misuse CSLM to evaluate NC penetration into spheroids, inevitably drawing misleading conclusions^63–72^. On the contrary, LSFM can readily image optically cleared spheroids up to 1 mm in diameter, albeit with lower lateral resolution compared to CLSM^56,57^. Our findings highlight the clear superiority of LSFM over CLSM in whole-spheroid imaging and demonstrate how LSFM can be used to predict whether a novel NC is likely to encounter uptake issues in solid tumors in vivo.

### Response of PDAC spheroids to chemotherapy

After confirming the uniform penetration of NCs in spheroids, we set out to study the chemotherapy response of our 3D models compared to that of 2D BxPC-3, PANC-1, and hPSC monocultures. We evaluated two different anti-PDAC drugs: gemcitabine (Gem), a nucleoside analog widely used in clinical PDAC management^23^; and SN-38, a potent yet difficult-to-formulate topoisomerase I inhibitor^73^ (Fig. S10). We assessed cell viability using CellTiter Glo®, a luminescence-based assay that quantifies adenosine triphosphate (ATP) levels following cell lysis. Common viability assays based on tetrazolium salts (e.g., MTT, MTS), which do not entail cell lysis, were deemed unsuitable for 3D culture due to possible limitations in the penetration of assay reagents into the spheroid core^33^.

In 2D, BxPC-3 and hPSC displayed similarly high sensitivity to both drugs, while PANC-1 was strongly drug-resistant (Fig. S11). This is consistent with the clinical observation that *KRAS*^*G12D*^-mutant tumors have a poorer response to treatment than wild-type *KRAS* tumors^74^. In 3D, spheroid viability did not follow a well-defined sigmoidal curve, hence no IC_50_ values could be determined. Instead, we compared the relative viabilities of corresponding 2D and 3D cultures at every drug concentration.

**Fig. 5:**
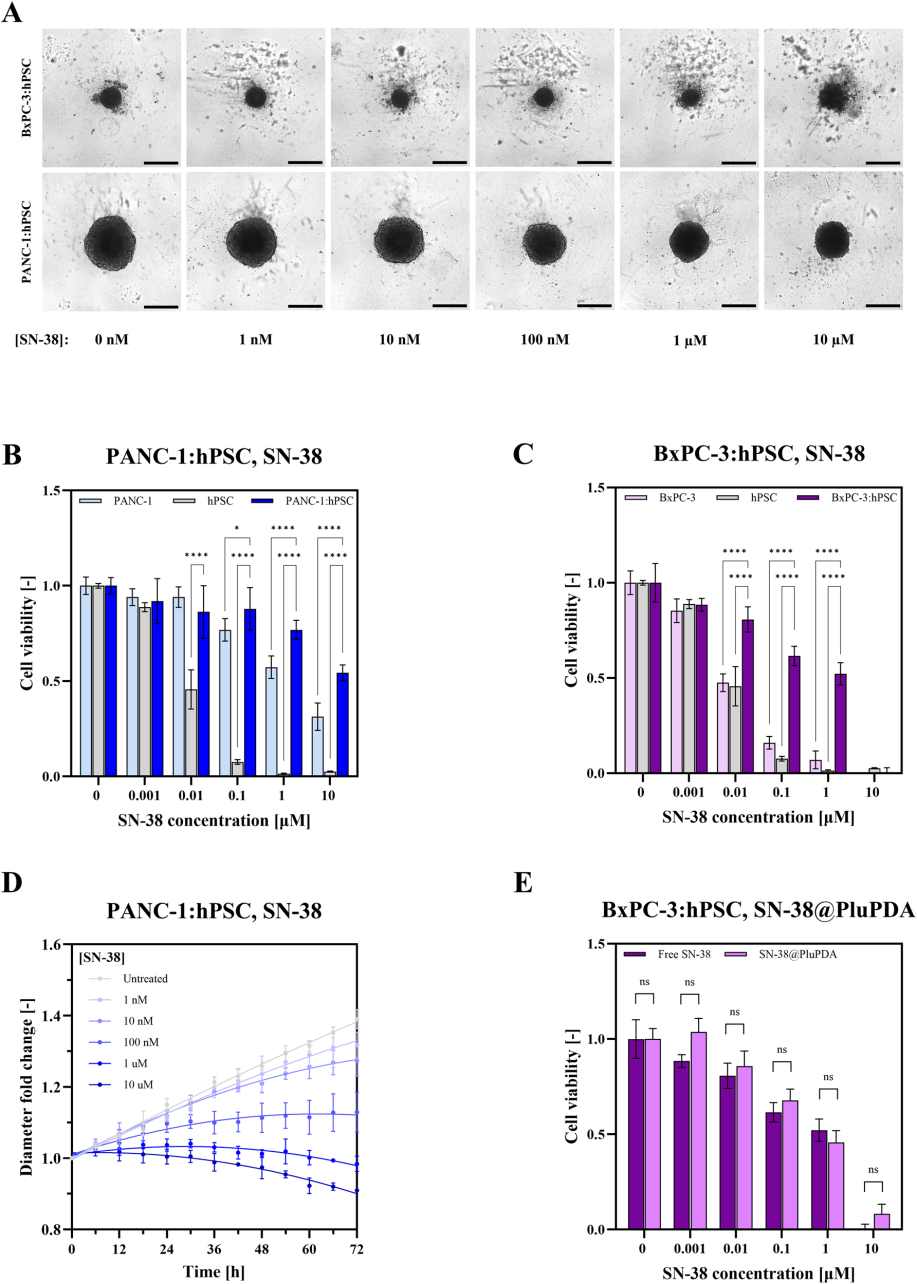
Spheroid response to chemotherapy. **A** Incucyte® images of BxPC-3:hPSC and PANC-1:hPSC spheroids after treatment with a range of SN-38 concentrations for 72 hours. Scale bars represent 500 µm. **B** Dose responses of 2D PANC-1 and hPSC monocultures and PANC-1:hPSC spheroids to SN-38. **C** Dose responses of 2D BxPC-3 and hPSC monocultures and BxPC-3:hPSC spheroids to SN-38. Viability was quantified using CellTiter Glo® and data were compared using two-way ANOVA. Statistically non-significant differences were not labeled. **D** Evolution of PANC-1:hPSC spheroid diameter over time following treatment with SN-38 calculated from Incucyte® images. **E** Dose response of BxPC-3:hPSC spheroids to free SN-38 and SN-38 encapsulated in PluPDA NCs (SN-38@PluPDA) quantified by CellTiter Glo®. Data were compared using two-way ANOVA.

Both spheroid models showed a significantly increased chemoresistance compared to their constituent cell lines cultured in 2D (Fig. 5A). PANC-1:hPSC spheroids were slightly more resistant to both drugs than 2D PANC-1 and vastly more resistant than 2D hPSC (Fig. 5B and S12A). For instance, 10 µM SN-38 reduced PANC-1:hPSC spheroid viability by 45.6% relative to control, whereas the same dose inhibited 2D PANC-1 by 68.7% and hPSC by 97.4%. Intriguingly, BxPC-3:hPSC spheroids also showed a high degree of chemoresistance despite being composed of two highly sensitive cell lines (Fig. 5C and S12B). For example, 1 µM SN-38 inhibited 2D BxPC-3 and hPSC cultures by 93.0% and 98.6% respectively while reducing BxPC-3:hPSC spheroid viability by just 47.8%. Notably, 10 µM SN-38 in BxPC-3:hPSC was the only condition that caused complete spheroid dissociation and loss of viability.

To complement the viability data, we performed a ToxiLight® cytotoxicity assay, which quantifies the extracellular release of adenylate kinase following cell death (Fig. S13). For most treatment conditions, only modest adenylate kinase release was detected despite significant reductions in viability, indicating that the effects of SN-38 and Gem were predominantly cytostatic rather than cytotoxic. In contrast, the treatment of BxPC-3:hPSC spheroids with 10 µM SN-38 resulted in extensive adenylate kinase release consistent with widespread cell death. Based on these findings, we recommend combining ATP-based viability assays with enzyme release-based cytotoxicity assays for more comprehensive characterization of different treatment options in spheroid models.

In addition to conventional reagent-based assays, it has been suggested that spheroid viability can be inferred through image analysis, greatly simplifying the experimental workflow^75,76^. Using Incucyte®, we observed a strong correlation (Pearson *r* = 0.9778) between spheroid diameter and CellTiter Glo®-based viability in PANC-1:hPSC (Fig. S14A). For this model, spheroid diameter can indeed serve as an accurate surrogate for viability, allowing for the real-time effects of a treatment to be observed instead of relying on endpoint viability measurements alone (Fig. 5D). However, for BxPC-3:hPSC, no correlation (Pearson r = 0.0580) was observed between diameter and viability (Supplementary S14B), with higher SN-38 concentrations resulting in increased debris production but no significant decrease in diameter (Fig. 5A). We therefore emphasize the need for caution when using spheroid size as a surrogate for viability. While this is useful for some models such as PANC-1:hPSC, it should never be done without validation *via* an alternative method.

The chemoresistance observed in spheroids was likely a product of multiple factors including pro-survival hPSC signaling^77^, cell adhesion-mediated drug resistance^78^ and, in the case of PANC-1:hPSC, hypoxia^51^. Although FN secretion by hPSCs has been implicated in Gem resistance^50^, our results show that the presence of hPSCs in BxPC-3 spheroids slightly increased their sensitivity to chemotherapy (Fig. S15), indicating that hPSCs remain susceptible to both Gem and SN-38 in 3D culture. In contrast, BxPC-3 cells appear to possess an inherent chemoresistance when cultured in 3D independently of hPSCs or hypoxia. Regardless of the underlying mechanisms, these highly drug-resistant models hold significant value for in vitro drug screenings. For instance, the relative inefficacy of Gem in our 3D models mirrors its modest clinical performance^23^, highlighting the need for more effective anti-PDAC therapeutics. Our model is well-suited to addressing this need due to its 96-well plate format, making it easily compatible with high-throughput screening of novel drug formulations.

Finally, we examined the effects of drug-loaded NCs on our spheroids, comparing them to treatment with a free drug. We employed 100 nm PluPDA NCs loaded with 6.8 wt% SN-38 (SN-38@PluPDA) (Fig. S5A, C–E) which have previously shown promise against PDAC cells in 2D^52^. In both spheroid models, SN-38@PluPDA showed no significant differences in growth inhibition compared to equivalent doses of free SN-38 (Fig. 5E and S16), as expected given the uniform penetration of PluPDA NCs into both spheroid types (Fig. 4C and S6B). Notably, PluPDA NCs showed significantly higher efficacy than equivalent concentrations of nab-paclitaxel (nab-PTX), a nanotherapeutic used to treat PDAC clinically, which inhibited spheroid growth by at most 30% (Fig. S17A). While a direct comparison between SN-38@PluPDA and nab-PTX is challenging due to differences in nanocarrier composition and active ingredient, the results nevertheless highlight the strong potential of SN-38@PluPDA as an anti-PDAC formulation. Importantly, NCs lacking SN-38 did not have any effect on spheroid viability, confirming their biocompatibility in a 3D context (Fig. S17B). Altogether, these data indicate that PluPDA NCs exhibit neither tissue penetration nor drug delivery limitations in 3D models, supporting their further evaluation in in vivo studies.

### Model application to lung adenocarcinoma

Finally, we sought to show that the protocols used to generate and characterize PANC-1:hPSC and BxPC-3:hPSC spheroids could be extended beyond PDAC. To this end, we produced lung cancer spheroids using A549 lung adenocarcinoma cells and WI-38 lung fibroblasts. With lung cancer remaining the leading cause of cancer-related death worldwide^79^, there is a pressing need for the development and clinical approval of new therapeutics, which can be facilitated by physiologically relevant high-throughput 3D spheroid models.

**Fig. 6:**
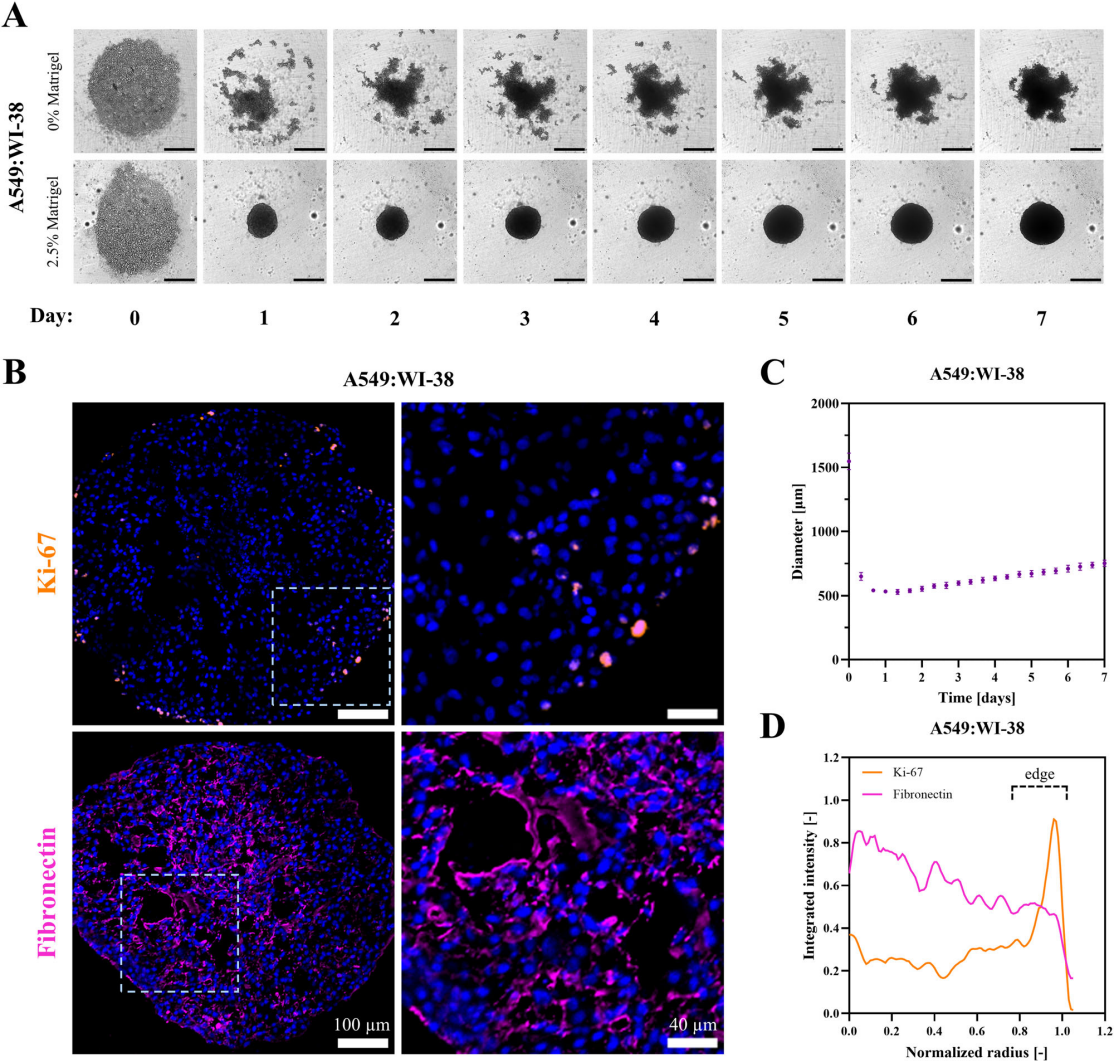
Characterization of the lung cancer spheroid model. **A** Incucyte® images of A549:WI-38 spheroids grown with and without supplementation with 2.5% Matrigel® for 7 days. Scale bars represent 500 µm. **B** Confocal microscopy images showing A549:WI-38 spheroid sections stained for proliferation (Ki-67 immunostaining, orange) and ECM deposition (fibronectin immunostaining, pink). **C** Evolution of spheroid diameter over time, calculated from Incucyte® images. **D** Integrated fluorescence intensity for all biomarkers across the entire spheroid section area for A549:WI-38, determined using the Radial Profile Extended plugin in ImageJ. Normalized radii of 0 and 1 represent the spheroid’s center and edge respectively.

A549:WI-38 spheroids exhibited size, morphology, and growth dynamics similar to those of PANC-1:hPSC (Fig. 6A, C, Supplementary Videos 13, 14), behaving as loose aggregates without ECM addition and forming dense steadily growing spheres when supplemented with 2.5% Matrigel®. They also possessed a comparable Ki-67 distribution to PANC-1:hPSC (Fig. 6B, D), suggesting that this feature is likely not cell line-specific and is caused by limitations in nutrient diffusion. However, unlike PANC-1:hPSC, A549:WI-38 developed a dense network of fibronectin throughout the entire spheroid, which would present a formidable barrier for NC penetration. Given that the stroma in general^80^ and fibronectin in particular^81,82^ are implicated in lung cancer progression, metastasis, and drug resistance, this model is likely to serve as an invaluable platform for the negative selection of novel therapeutics. More generally, the ease with which our PDAC model was adapted to lung cancer suggests that it can be further tailored to many other cancer types on demand, giving it a myriad of possible applications.

## Discussion

In this study, we present a well-characterized platform for the generation of 3D spheroid co-cultures comprising cancer and stromal cells. We focus primarily on PDAC, and our model successfully recapitulates several aspects of this disease which are difficult to study in 2D, including hypoxia, fibrosis and chemoresistance. In particular, our spheroids are significantly resistant to Gem and SN-38 even when composed of cells that are highly sensitive to those drugs in 2D. Additionally, we use our model to show that PluPDA NCs exhibit excellent tissue penetration and can effectively deliver SN-38 to cancer cells in 3D, suggesting that these NCs are ready for in vivo trials. Notably, we demonstrate that confocal microscopy is inadequate for studying NC penetration in 3D and recommend LSFM as a viable alternative.

Our spheroid model strives to be easily reproducible by other researchers, requiring no specialized equipment. The model is also highly modular, and our methods can be readily adapted to other malignancies, exemplified by our simple generation of A549:WI-38 lung cancer spheroids. To further increase our model’s physiological accuracy, it may be modified in the future to incorporate other cell types, such as immune and/or endothelial cells. Ultimately, the widespread adoption of our model in pre-clinical nanomedicine research and its possible extension to clinical precision medicine would fill the gap between overly simplistic 2D cell culture and resource-intensive animal studies, leading to a more efficient translation pathway.

## Methods

### Materials

All materials used in this study were purchased from Thermo Fisher Scientific, Merck, VWR, Abcam, Biorbyt, Cambridge Bioscience, Fluorochem, Hypoxyprobe, Ibidi, Medicell Membranes, MP Biomedicals, Promega, or Proteintech and used without further purification. All water used was Milli-Q ultrapure grade. A detailed list of materials, suppliers, and catalogue references is available in the Supplementary Methods.

### General cell culture

The human cell lines A549, BxPC-3, PANC-1 and WI-38 were purchased from the American Type Culture Collection (ATCC). SV40-immortalized human pancreatic stellate cells (hPSC) were a gift from Dr Giulia Biffi (Cancer Research UK Cambridge Institute). PANC-1, A549 and WI-38 cells were cultured in DMEM supplemented with 10% FBS. BxPC-3 cells were cultured in RPMI 1640 supplemented with 10% FBS. hPSC cells were grown in DMEM supplemented with 5% FBS. All culture media were additionally supplemented with 1% penicillin-streptomycin. All cells were cultured at 37 °C, 5% CO_2_ in a humidified atmosphere, passaged at sub-80% confluence using TrypLE Express, and not allowed to exceed passage number 20.

### Spheroid culture

Cancer and stromal cells were seeded into ultra-low-attachment round-bottom 96-well plates in 100 µL of complete cell culture medium per well. Cells were seeded in appropriate mixtures of the media used for their monoculture: for example, BxPC-3:hPSC(1:1) spheroids were seeded in a 1:1 mixture of RPMI 1640 + 10% FBS and DMEM + 5% FBS. A 1.25—10% stock solution of Matrigel® in complete medium was prepared on ice and 100 µL of this stock were added to each well, followed by gentle pipette mixing. For collagen experiments, the Matrigel® was replaced with 30—120 µg/mL of collagen type I. For matrix-free experiments, 100 µL of pure complete media were added instead. The cells were allowed to settle at room temperature for 30 minutes, then the plates were centrifuged at 800 RCF, room temperature for 15 minutes. The resulting spheroids were cultured under standard conditions and their growth was monitored using an Incucyte® S3 Live-Cell Analysis System (Sartorius). Spheroid circularity and aspect ratio were calculated from Incucyte® images as follows:

$$\mathrm{Circularity}\left[-\right]=\frac{4{\rm{\pi }}\times \mathrm{Spheroid\; area}\,[{{\rm{\mu }}{\rm{m}}}^{2}]}{(\mathrm{Spheroid\; perimeter}\,[{{\rm{\mu }}{\rm{m}}}^{2}])}$$

$$\mathrm{Aspect\; ratio}\left[-\right]=\frac{\mathrm{Spheroid\; major\; axis\; length}\,[{\rm{\mu }}{\rm{m}}]}{\mathrm{Spheroid\; minor\; axis\; length}\,[{\rm{\mu }}{\rm{m}}]}$$

### Scanning electron microscopy

Spheroids were washed twice with PBS, then fixed using a solution of 4% PFA and 1% glutaraldehyde in PBS for 2 hours at room temperature. Following fixation, the spheroids were dehydrated using ethanol solutions of increasing concentration, spending 15 minutes in each of 30%, 50%, 85%, 95%, 100% and then 100% ethanol again. Finally, the spheroids were dried at 60 °C for 20 minutes, coated with a 10 nm platinum film using a Q150T ES Turbo-Pumped Sputter Coater (Quorum Technologies), and imaged using a MIRA3 FEG-SEM microscope (TESCAN) operated at 5 kV.

### Spheroid cryosectioning

**Fig. 7:**
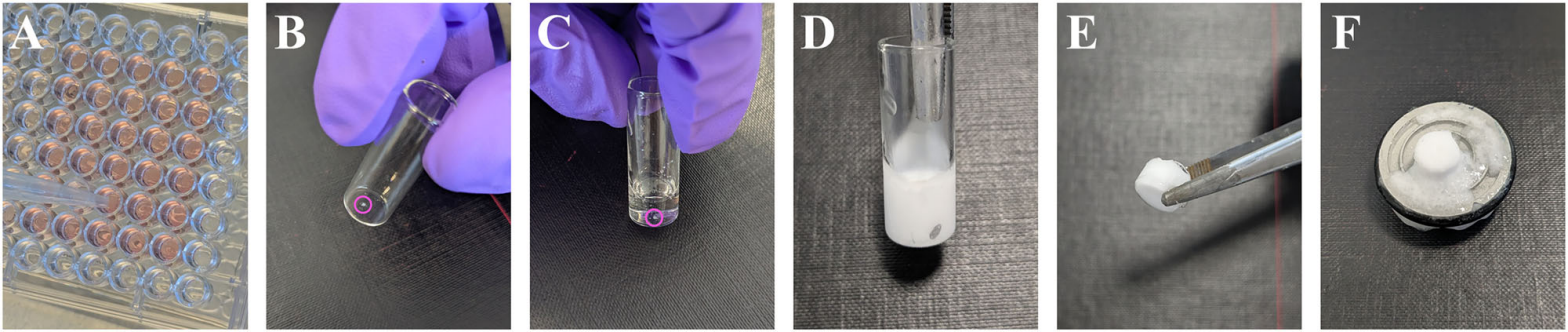
Visual aid for preparing spheroids for sectioning. **A** A spheroid is removed from its 96-well plate using a 1 mL pipette tip with the bottom ~ 5 mm cut off to increase its diameter. **B** The spheroid after transfer to a cylindrical glass vial and medium removal. **C** The spheroid submerged under ~ 1 cm of OCT compound. **D** The spheroid after freezing in liquid nitrogen for 30 seconds. The side of the glass vial was marked to facilitate finding the spheroid during sectioning. **E** The block of OCT after being pulled out of the vial with tweezers. **F** The block of OCT attached to a cryostat chuck using a drop of OCT, then briefly solidified at −20 °C.

Spheroids were transferred to cylindrical glass vials and washed twice with PBS, then fixed using 4% PFA in PBS for 2 hours at room temperature. Following fixation, they were washed twice with PBS and incubated overnight in a solution of 30% w/v sucrose in PBS at 4 °C on an orbital shaker. The next day, the spheroids were again washed twice with PBS and embedded in OCT compound, then immediately snap-frozen in liquid nitrogen and stored at −80 °C. For sectioning, a CM1950 CryoStat (Leica) operated at −20 °C was employed to obtain 10 µm-thick sections, which were then mounted on glass microscopy slides and stored at −20 °C. A visual aid is provided in Fig. 7:

### Immunofluorescence

Spheroid sections mounted on slides were permeabilized using 0.3% Triton X-100 in PBS for 15 minutes, then blocked using 1% w/v BSA in PBS for 1 hour at room temperature. Subsequently, the sections were incubated overnight with rabbit anti-human primary antibodies diluted in blocking solution (1:500 for Ki-67, 1:150 for fibronectin) at 4 °C. The following day, the sections were washed 3 times with PBS, then incubated with a fluorescently labeled goat anti-rabbit secondary antibody diluted in PBS (1:1000) for 1 hour at room temperature in the dark. Afterwards, they were again washed 3 times with PBS, then incubated with 0.1 µg/ml of DAPI for 5 minutes at room temperature in the dark. Finally, the sections were mounted under a coverslip using ProLong^TM^ Diamond Antifade Mountant and left to dry in the dark for 5 minutes before being imaged by an Axio Observer Z1 LSM 800 confocal microscope (Zeiss). Images were analyzed using ZEN 3.5 (Zeiss) and ImageJ^83^.

### Hypoxyprobe™ assay

The Hypoxyprobe™ Kit—Rat MAb was used to visualize hypoxic cells according to the manufacturer’s instructions. Briefly, live spheroids were incubated with 200 µM pimonidazole hydrochloride in complete culture medium at 37 °C overnight. The spheroids were then fixed, sectioned, permeabilized, and blocked as for immunofluorescence, then stained using a rat anti-human pimonidazole antibody diluted in blocking solution (1:1000). The following day, the sections were washed 3 times with PBS, then incubated with a fluorescently labeled goat anti-rat secondary antibody diluted in PBS (1:1000) for 1 hour at room temperature in the dark. The sections were then washed, stained with DAPI, mounted and imaged.

### TUNEL assay

Spheroid sections were stained for late-stage apoptosis using a CoraLite® Plus 488 TUNEL Assay Apoptosis Detection Kit (green) according to the manufacturer’s instructions. Briefly, fixed spheroid sections were permeabilized using 0.3% Triton X-100 in PBS for 15 minutes, then each section was incubated with 1 µL terminal deoxynucleotidyl transferase (TdT) solution in 50 µL of TUNEL reaction buffer for 2 h at 37 °C. The sections were then washed 3 times with PBS and stained with DAPI, mounted and imaged in the same way as immunofluorescence sections.

### Spheroid section image analysis

Plots of normalized fluorescence intensity against normalized radius were generated using the Radial Profile Extended plugin in ImageJ, which integrates fluorescence intensity and presents it as a function of radius. To account for the spheroid sections not being perfectly circular, each image was partitioned into 12 30 ° segments, which were integrated separately. In each segment, radial distance was normalized to the distance between the segment’s center and edge, as determined from DAPI counterstaining. These data were then averaged, normalized to each image’s maximum intensity and smoothed to a 5-point moving average using Prism 10 (GraphPad).

### Whole-spheroid imaging

Live 3-day-old spheroids were incubated with 50 µg/ml rhodamine B-labeled 100 nm polydopamine-Pluronic® F127 (PluPDA) NCs in complete culture medium for 24 hours (details on NC synthesis and labelling are available in the Supplementary Methods). The spheroids were then washed twice with PBS, fixed using 4% PFA in PBS for 2 hours at room temperature and optically cleared using the Sca*l*eA2 method^53^ by incubating in a solution of 8 M urea, 10% glycerol and 0.1% Triton X-100 in PBS for 72 hours at 4 °C. The spheroids were again washed twice with PBS, then their cell nuclei were stained with 0.5 µM SYTOX™ Green in PBS for 72 hours at 4 °C. Finally, the spheroids were again washed twice with PBS, mounted in the center of a glass-bottom dish inside a 20 µL droplet of 1% agarose and imaged using both an Axio Observer Z1 LSM 800 confocal microscope (Zeiss) and a custom-built light sheet microscope. The latter was based on a modified inverted selective plane illumination microscopy (iSPIM) design^84^ equipped with water-immersion Nikon Plan Fluor 10 × 0.3 NA illumination and a Zeiss 20×0.5 NA detection objectives and a Hamamatsu ORCA-Flash 4.0 camera, with stage-scanned acquisition implemented using components from Applied Scientific Instrumentation (ASI) and controlled by Micro-Manager software using the diSPIM plugin from ASI. Images were analyzed using ZEN 3.5 and ImageJ. Fluorescence intensity distributions across every optical section were analyzed as described in “Spheroid section image analysis”.

### Viability studies

For 2D studies, all cell lines were seeded into 96-well flat-bottom plates at 3000 cells per well and allowed to attach overnight. Medium was then fully aspirated, and the cells were dosed with a range of gemcitabine and SN-38 concentrations (1 nM–10 µM) in 100 µL of complete medium containing 0.5% DMSO. After 72 hours, the cells were assayed for viability using CellTiter Glo® according to the manufacturer’s protocol. Briefly, 100 µL of assay reagent were added to every well and incubated at room temperature for 10 minutes. The contents of all wells were then transferred to a white 96-well plate and luminescence was recorded using a CLARIOstar^Plus^ plate reader (BMG Labtech).

For 3D studies, 3-day-old spheroids had their medium fully aspirated, followed by dosing with the same range of gemcitabine and SN-38 concentrations as the 2D cells. After 72 hours of incubation, spheroid viability was assayed using CellTiter Glo®. To induce complete cell lysis in spheroids, they were incubated with the assay reagent for 45 minutes with vigorous pipette-mixing every 15 minutes.

For drug-loaded NC studies, 100 nm PluPDA NCs loaded with 6.8 wt% SN-38 were used (details on NC drug loading are available in the Supplementary Methods). 3-day-old spheroids were dosed with 5.8 ng/mL—57.6 µg/mL SN-38@PluPDA NCs, corresponding to 1 nM–10 µM SN-38. After 72 hours of incubation, spheroid viability was assayed using CellTiter Glo®. For control experiments, spheroids were dosed with 5.8 ng/mL—57.6 µg/mL drug-free PluPDA or nab-paclitaxel.

### Cytotoxicity studies

Spheroids were dosed with drugs as described in “Viability studies”. After 72 hours of incubation, 20 µL of the supernatant from each spheroid were transferred into a white 96-well plate, mixed with 100 µL of ToxiLight® assay reagent, incubated at room temperature in the dark for 10 minutes, then measured for luminescence using a CLARIOstar^Plus^ plate reader (BMG Labtech). As a maximum cytotoxicity control, spheroids were incubated with 0.5% Triton X-100 in complete media for 30 mins to induce lysis.

### Statistical analysis

Unless otherwise specified, experiments were conducted in independent triplicate, with all data presented as mean ± standard deviation. Statistical analyses were performed in Prism 10 (GraphPad). Statistical significance levels were denoted as follows: ns for p > 0.05, * for p ≤ 0.05, ** for p ≤ 0.01, *** for p ≤ 0.001 and **** for p ≤ 0.0001.

## Supplementary information

Supplementary information

Supplementary video1

Supplementary video2

Supplementary video3

Supplementary video4

Supplementary video5

Supplementary video6

Supplementary video7

Supplementary video8

Supplementary video9

Supplementary video10

Supplementary video11

Supplementary video12

Supplementary video13

Supplementary video14

## Data Availability

Data is provided within the manuscript or supplementary information files and videos.
