# Supplementary material for: A 3D spheroid model for assessing nanocarrier-based drug delivery to solid tumors
Source: NPJ Biomed Innov. 2025 Oct 24;2:37. doi: 10.1038/s44385-025-00041-x (PMC12552133; doi:10.1038/s44385-025-00041-x)
Supplement: Supplementary file 1 — Supplementary information [file 44385_2025_41_MOESM1_ESM.pdf]

Supplementary Material

for

A 3D Spheroid Model for Assessing Nanocarrier-based Drug Delivery to Solid Tumors

Chitra Yadav[†]*, Alexander S. Evtushenko*[†]*, Andrea Bistrović Popov, Beatriz Lozano Torres, Ishtiaq Ahmed, Liuba Dvinskikh, Clemens F. Kaminski, Ljiljana Fruk**

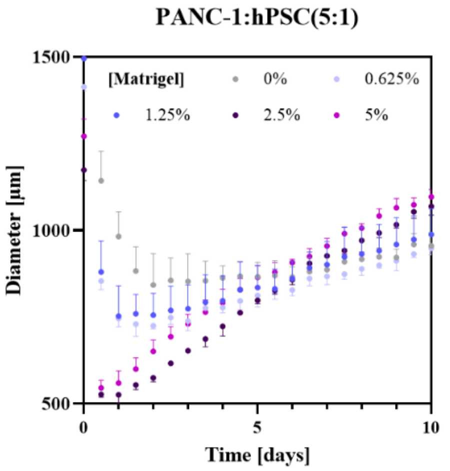

Figure S1. *Effect of Matrigel® concentration on PANC-1:hPSC(5:1) spheroid growth dynamics calculated from Incucyte® images.*

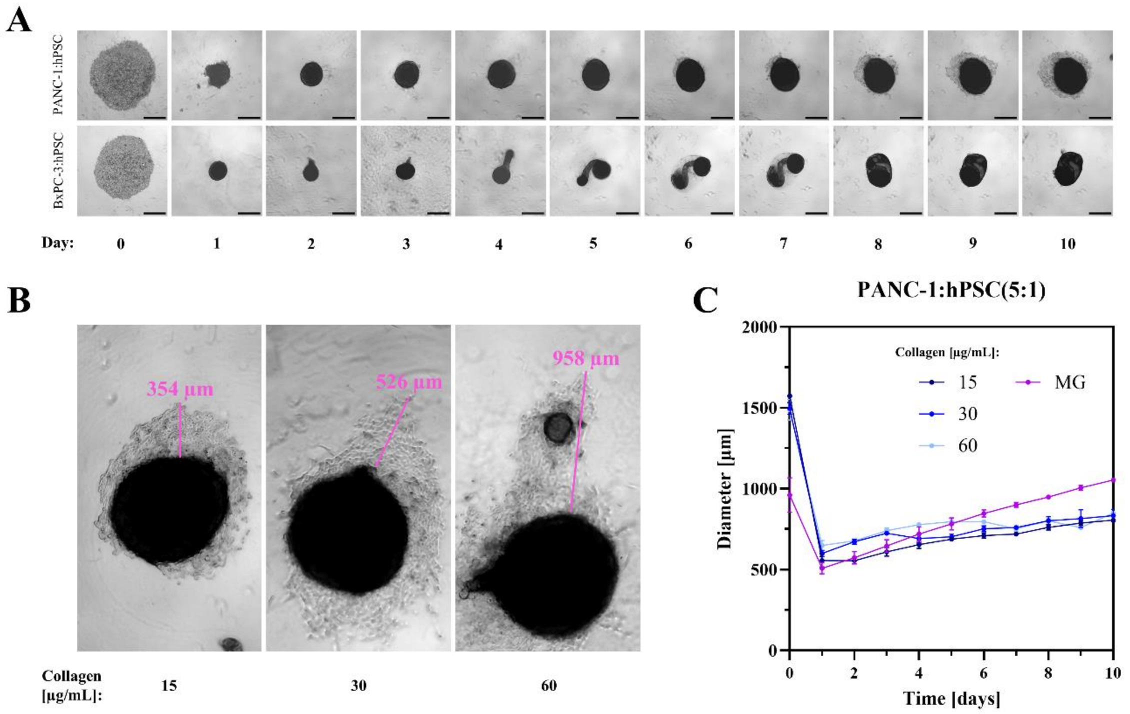

Figure S2. A) *Incucyte® images of PANC-1:hPSC(5:1) and BxPC-3:hPSC(5:1) spheroids grown in media supplemented with 15 µg/mL collagen I for 10 days. Scale bars represent 500 µm.* ***B)*** *Incucyte® images of PANC-1:hPSC(5:1) spheroids supplemented with 15, 30, and 60 µg/mL collagen I showing the extent of invasion on day 10.* ***C)*** *Effect of collagen I concentration on PANC-1:hPSC(5:1) spheroid growth dynamics compared to 2.5% Matrigel® (MG) calculated from Incucyte® images.*

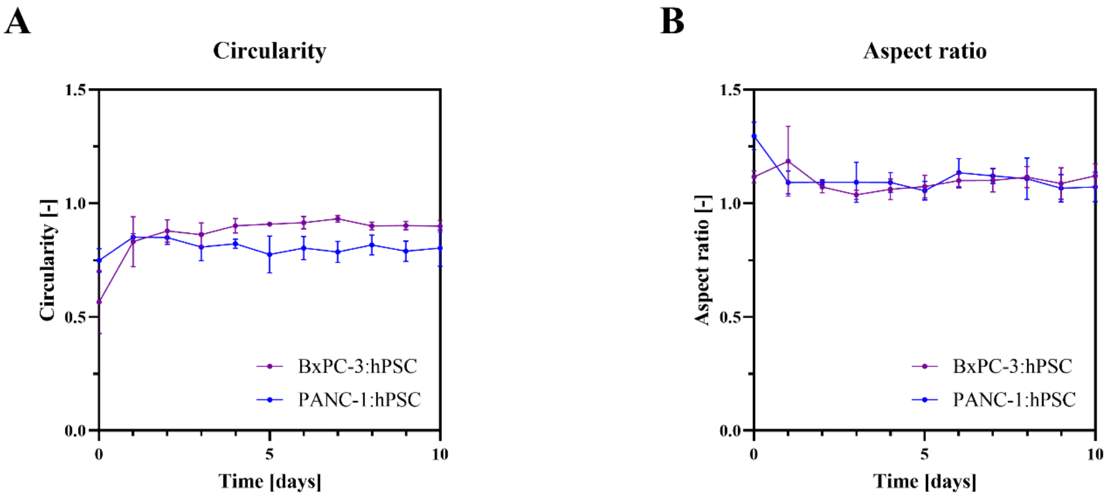

Figure S3. A) *Evolution of BxPC-3:hPSC and PANC-1:hPSC spheroid circularity and* ***B)*** *aspect ratio over time as calculated from Incucyte® images.*

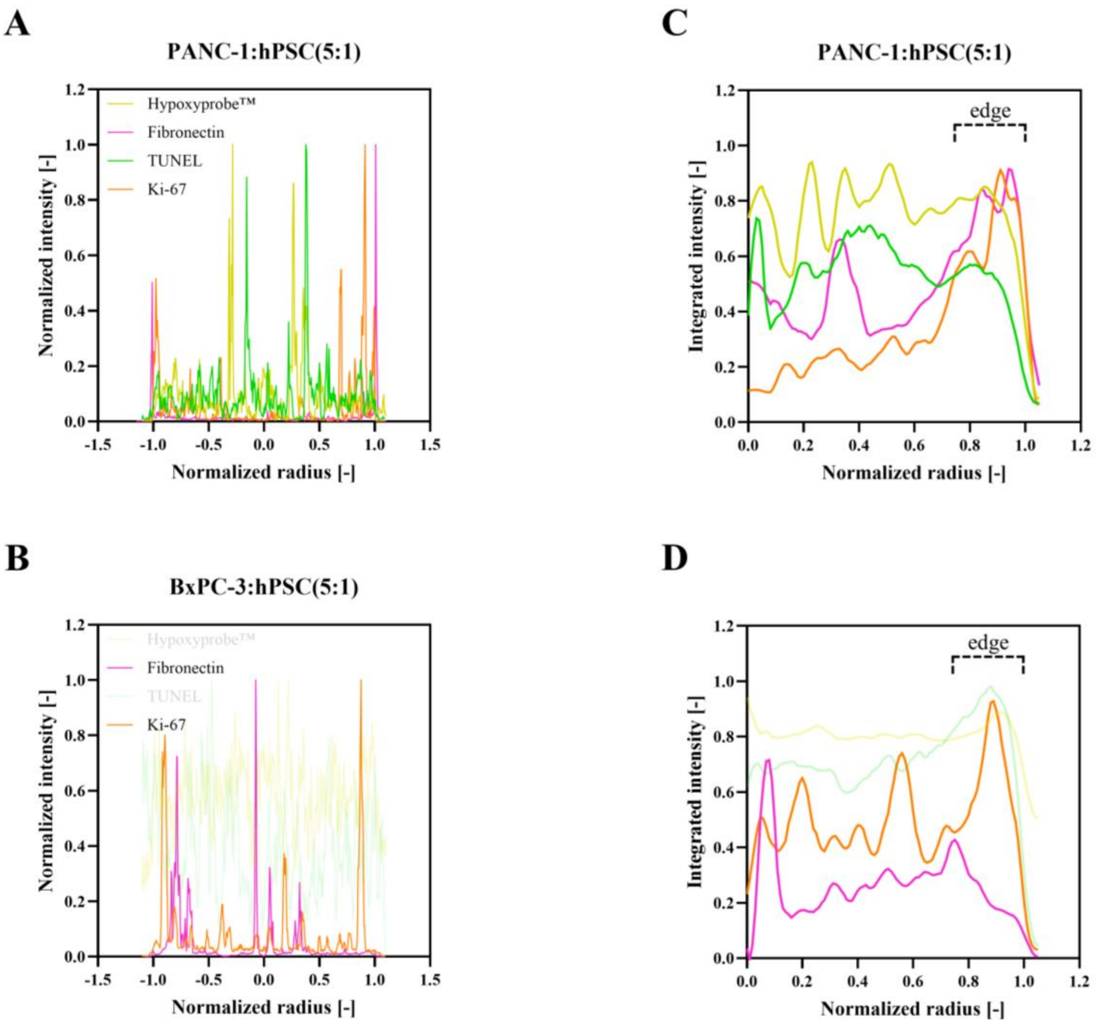

Figure S4. A) *Fluorescence intensity distribution for all biomarkers along a straight line drawn across PANC-1:hPSC(5:1) and* ***B)*** *BxPC-3:hPSC(5:1) spheroid sections. A normalized radius of 0 represents the spheroid's center, while +1 and –1 represent opposite edges.* ***C)*** *Integrated fluorescence intensity for all biomarkers across the entire spheroid section area for PANC-1:hPSC(5:1) and* ***D)*** *BxPC-3:hPSC(5:1), determined using the Radial Profile Extended plugin in ImageJ. Normalized radii of 0 and 1 represent the spheroid's center and edge respectively.*

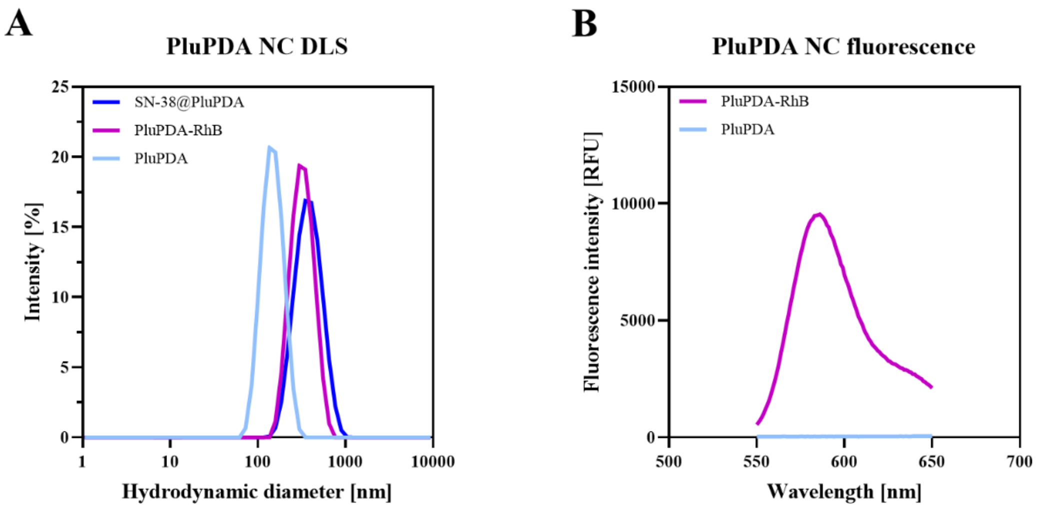

C

Nanocarrier	Z-average diameter [nm]	PDI [-]
PluPDA	119.0 ± 1.0	0.091 ± 0.015
PluPDA-RhB	136.3 ± 3.3	0.126 ± 0.027
SN-38@PluPDA	145.6 ± 3.5	0.005 ± 0.001

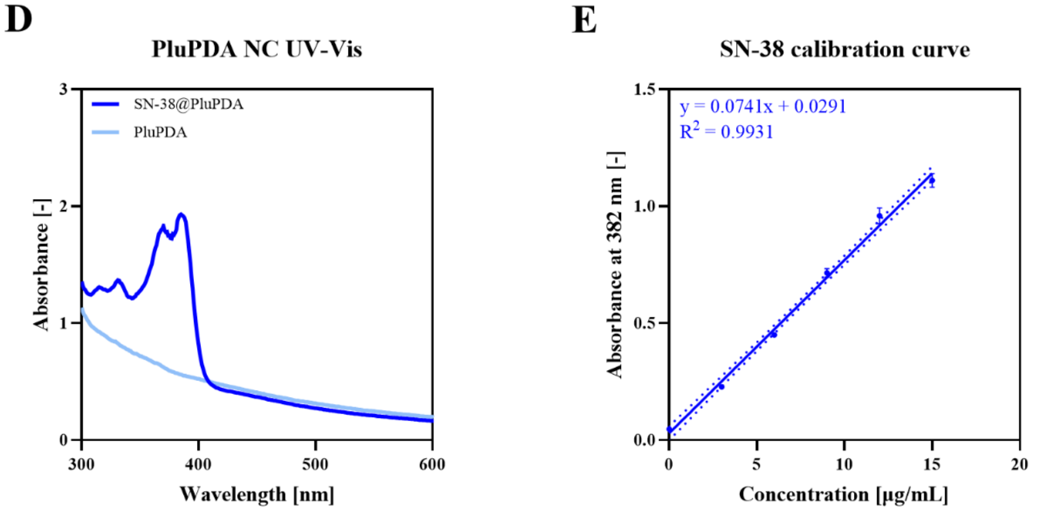

Figure S5. *Characterization of the nanocarriers used in this study.* ***A)*** *Dynamic light scattering (DLS) intensity distributions of unmodified polydopamine-Pluronic® F127 NCs (PluPDA), PluPDA NCs covalently labelled with rhodamine B (PluPDA-RhB), and PluPDA NCs loaded with SN-38 (SN-38@PluPDA).* ***B)*** *Fluorescence emission spectra of PluPDA and PluPDA-RhB excited at 530 nm showing the successful attachment of rhodamine B to the NCs* ***C)*** *Summary of the hydrodynamic diameter and polydispersity index (PDI) values of the different NCs.* ***D)*** *UV-Vis absorbance spectra of PluPDA and SN-38@PluPDA showing the successful loading of SN-38.* ***E)*** *Calibration curve used to determine SN-38 loading. The dashed lines represent the 95% confidence interval of the fit.*

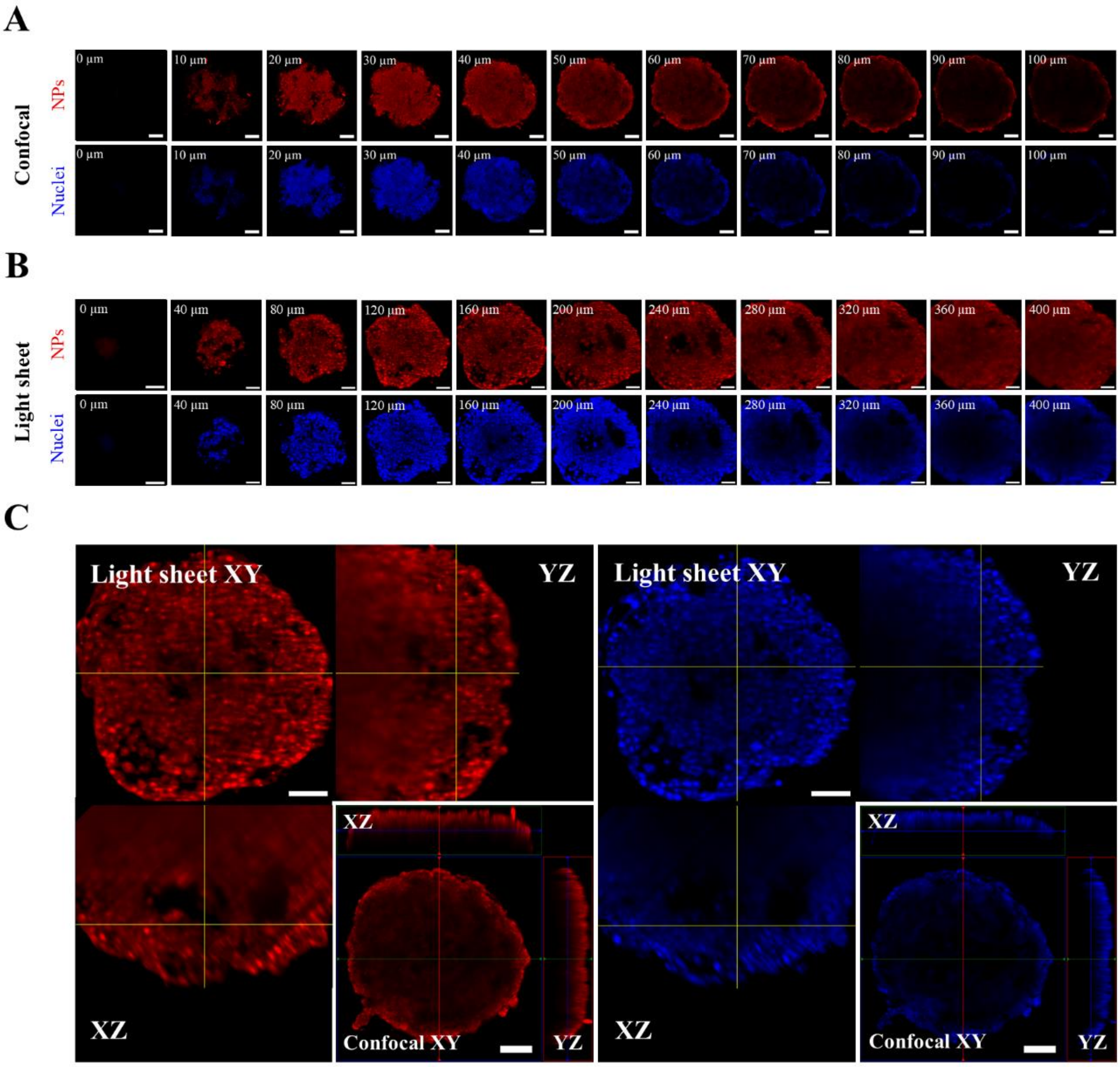

Figure S6. A) *Z-stack confocal laser scanning microscopy images of a PANC-1:hPSC spheroid incubated with rhodamine B-labeled polydopamine-Pluronic® F127 NCs. Optical sections are shown for every 10 µm up to a depth of 100 µm.* ***B)*** *Light sheet fluorescence microscopy images of the same spheroid. Optical sections are shown for every 40 µm up to a depth of 400 µm.* ***C)*** *Orthogonal slices of the whole spheroid generated for both techniques. In all images, NCs are shown in red and cell nuclei, stained with SYTOX™ Green, are shown in blue. All scale bars represent 100 µm.*

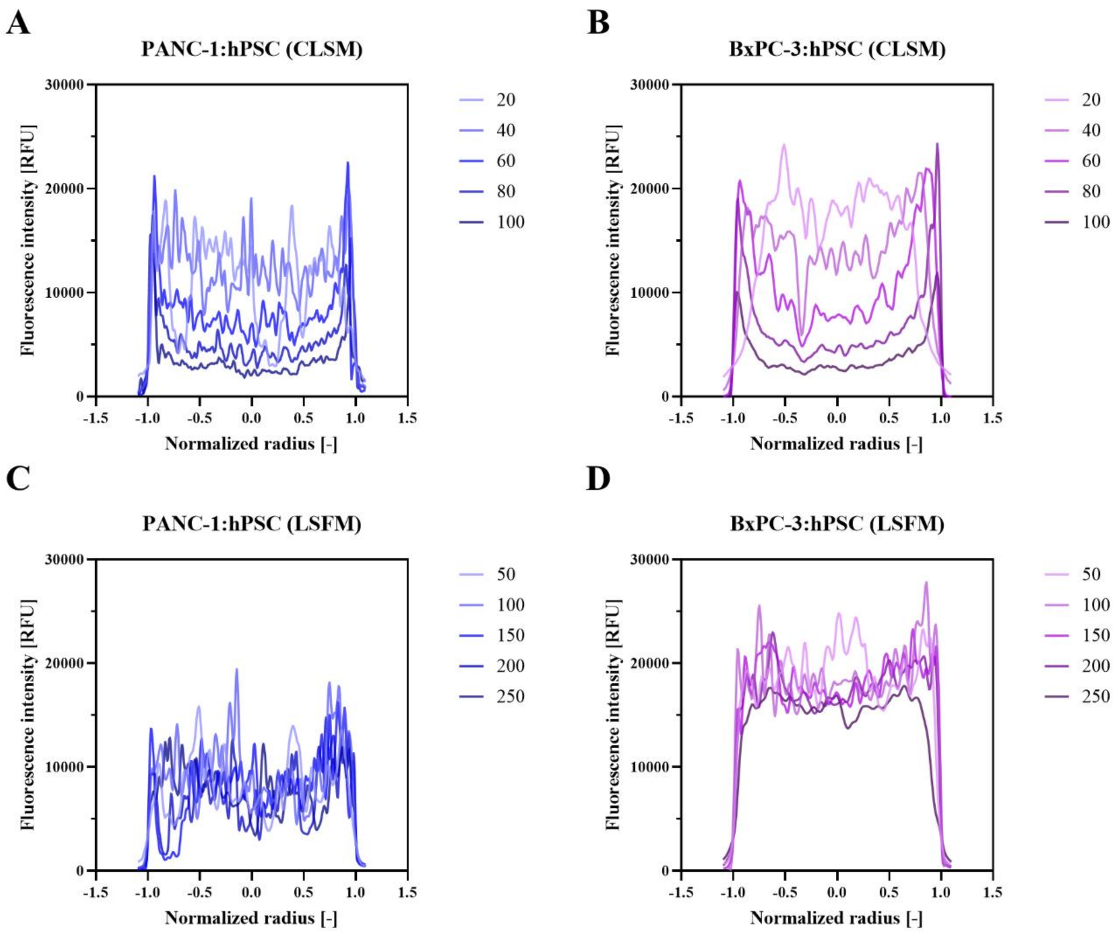

Figure S7. A) *Fluorescence intensity profiles of rhodamine B-labelled polydopamine-Pluronic® F127 NCs along a straight line drawn across PANC-1:hPSC(5:1) and* ***B)*** *BxPC-3:hPSC(5:1) spheroids imaged using confocal laser scanning microscopy (CLSM) at different depths. A normalized radius of 0 represents the spheroid's center, while +1 and –1 represent opposite edges.* ***C)*** *and* ***D)*** *Similar profiles obtained using light sheet fluorescence microscopy (LSFM).*

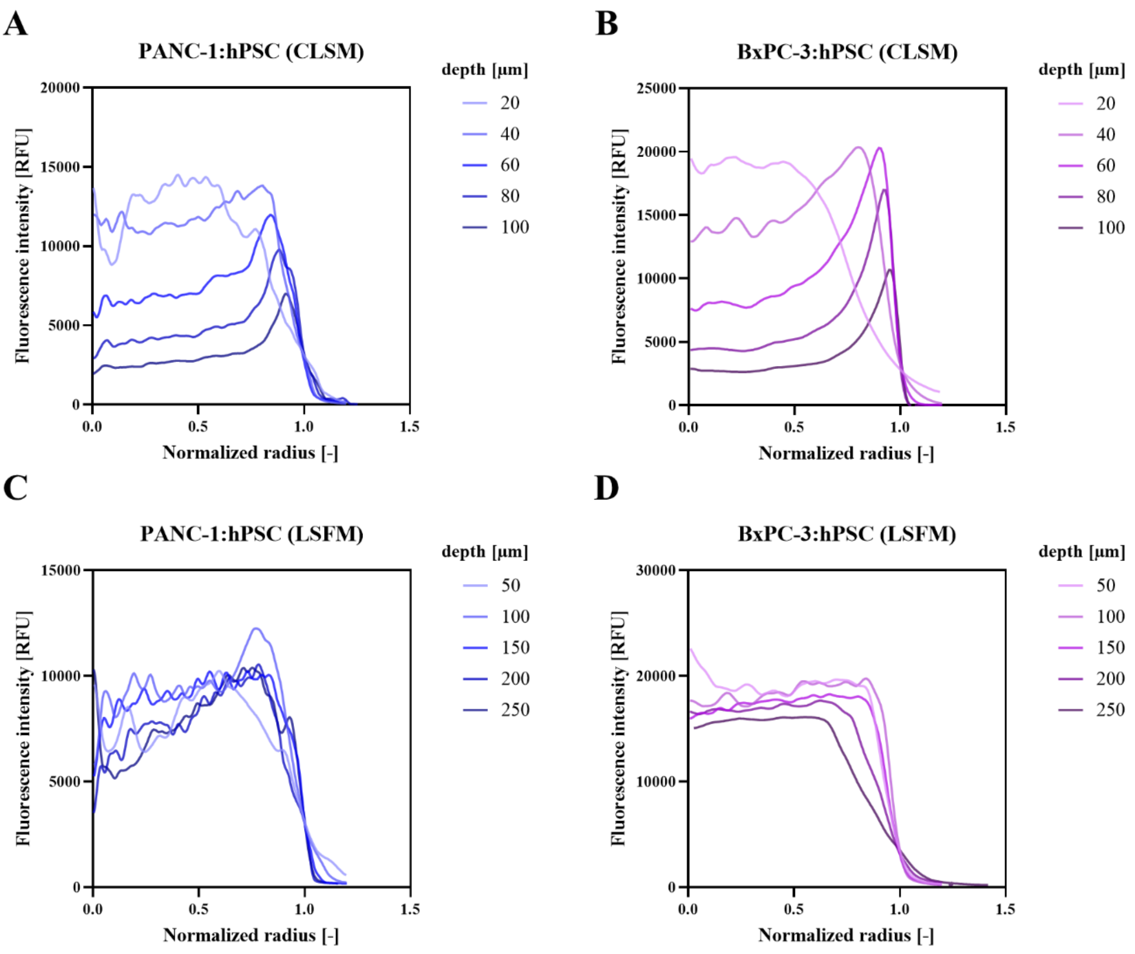

Figure S8. A) *Fluorescence intensity profiles of rhodamine B-labelled polydopamine-Pluronic® F127 NCs integrated across the entire PANC-1:hPSC(5:1) and* ***B)*** *BxPC-3:hPSC(5:1) spheroid area based on confocal laser scanning microscopy (CLSM) imaging at different depths. Integration was performed using the Radial Profile Extended plugin in ImageJ. Normalized radii of 0 and 1 represent the spheroid's center and edge respectively.* ***C)*** *and* ***D)*** *Similar profiles obtained using light sheet fluorescence microscopy (LSFM).*

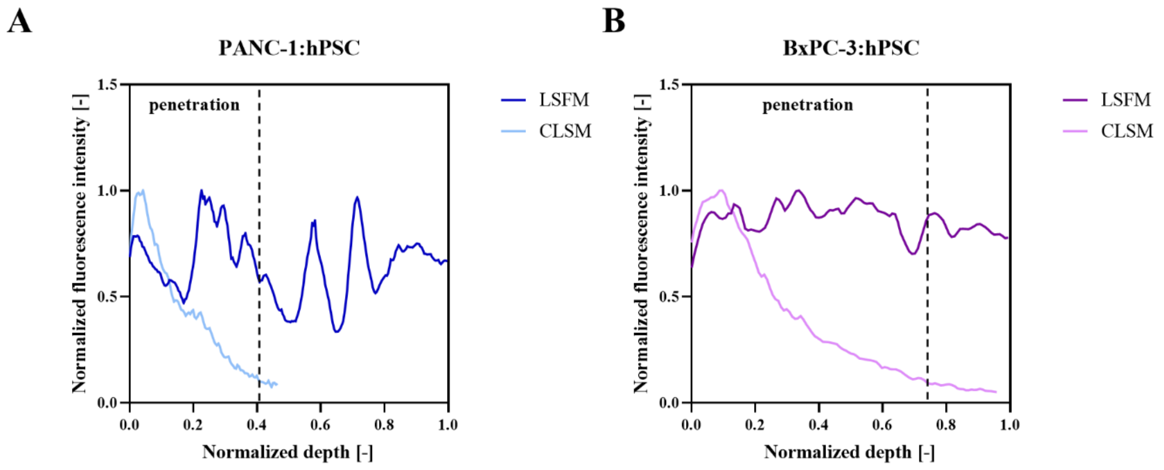

Figure S9. A) *Fluorescence intensity profiles of rhodamine B-labelled polydopamine-Pluronic® F127 NCs at a fixed point in the x-y plane as a function of depth through PANC-1:hPSC(5:1) and* ***B)*** *BxPC-3:hPSC(5:1) spheroids imaged using both confocal laser scanning microscopy (CLSM) and light sheet fluorescence microscopy (LSFM). Normalized depths of 0 and 1 represent the spheroid's edge and center respectively. Dashed lines indicate the limit of NC penetration into the spheroids as predicted by CLSM, defined as the depth at which normalized fluorescence intensity falls below 0.1.*

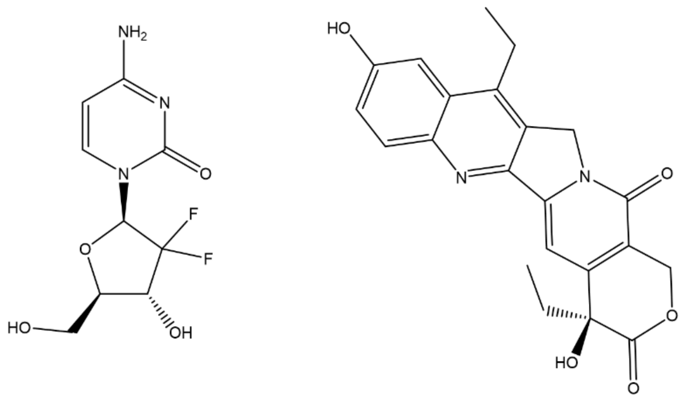

Gemcitabine **SN-38**

Figure S10. *Chemical structures of gemcitabine and SN-38.*

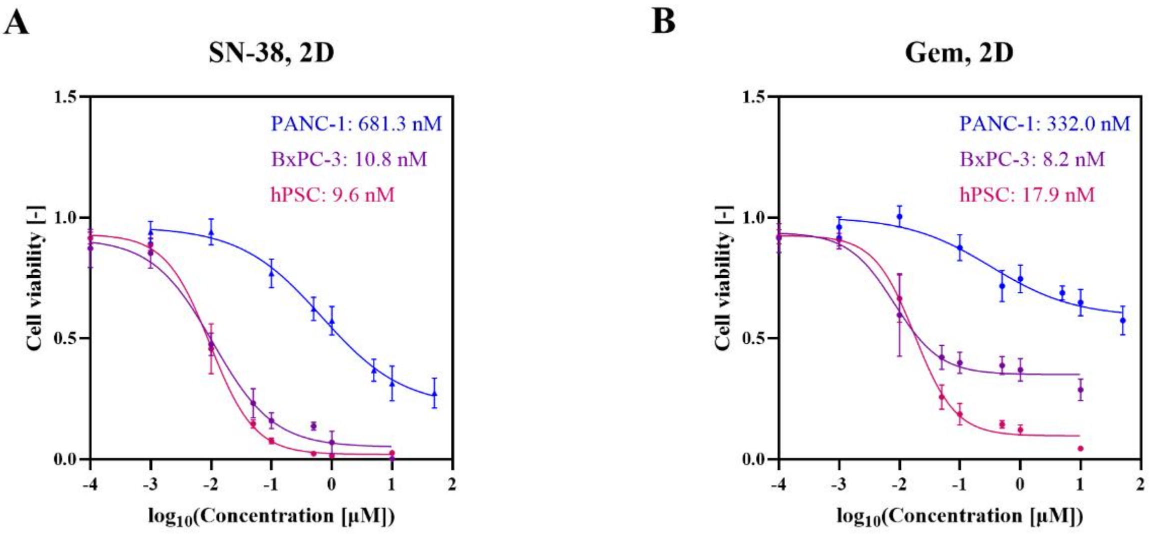

Figure S11. *Dose responses of 2D PANC-1, BxPC-3, and hPSC monocultures to* ***A)*** *SN-38 and* ***B)*** *gemcitabine quantified by CellTiter Glo®. IC_{50} values are shown on each plot.*

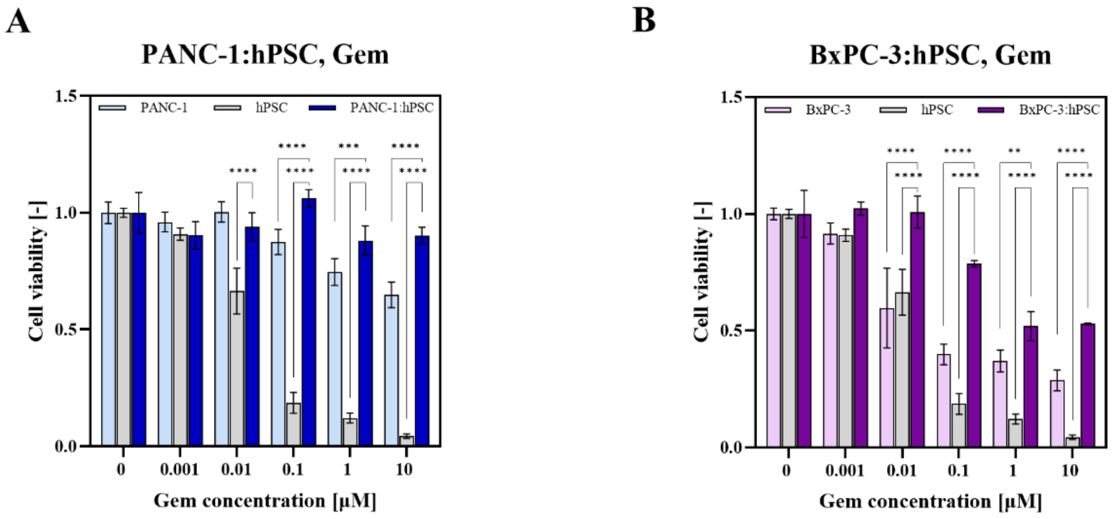

Figure S12. A) *Dose responses of 2D PANC-1 and hPSC monocultures and PANC-1:hPSC spheroids to gemcitabine.* ***B)*** *Dose responses of 2D BxPC-3 and hPSC monocultures and BxPC-3:hPSC spheroids to gemcitabine. Viability was quantified using CellTiter Glo® and data were compared using two-way ANOVA.*

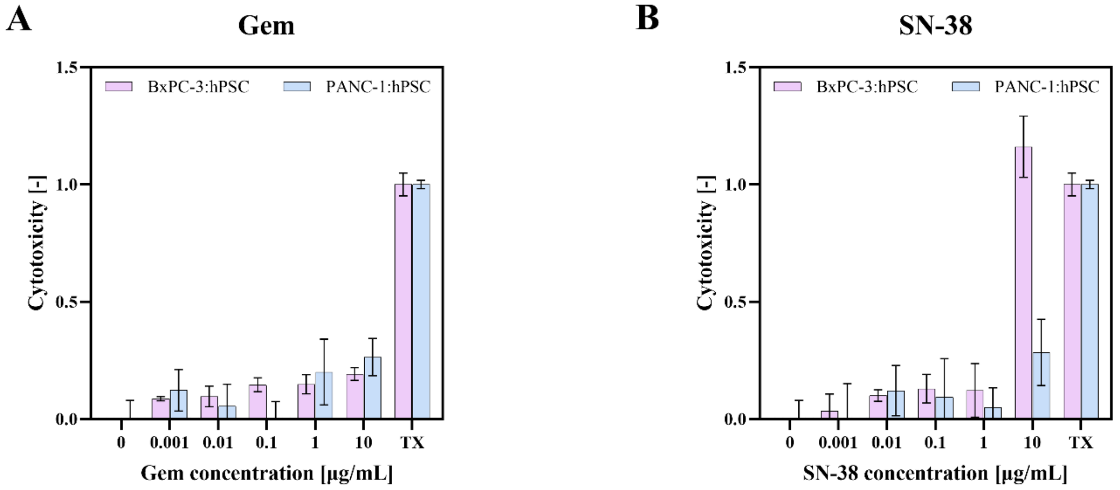

Figure S13. *Cytotoxicity induced in BxPC-3:hPSC and PANC-1:hPSC spheroids following treatment with* ***A)*** *SN-38 and* ***B)*** *gemcitabine as measured using a ToxiLight® adenylate kinase release assay. As a maximum cytotoxicity control, spheroids were incubated with 0.5% Triton X-100 (TX) in complete media for 30 mins to induce lysis.*

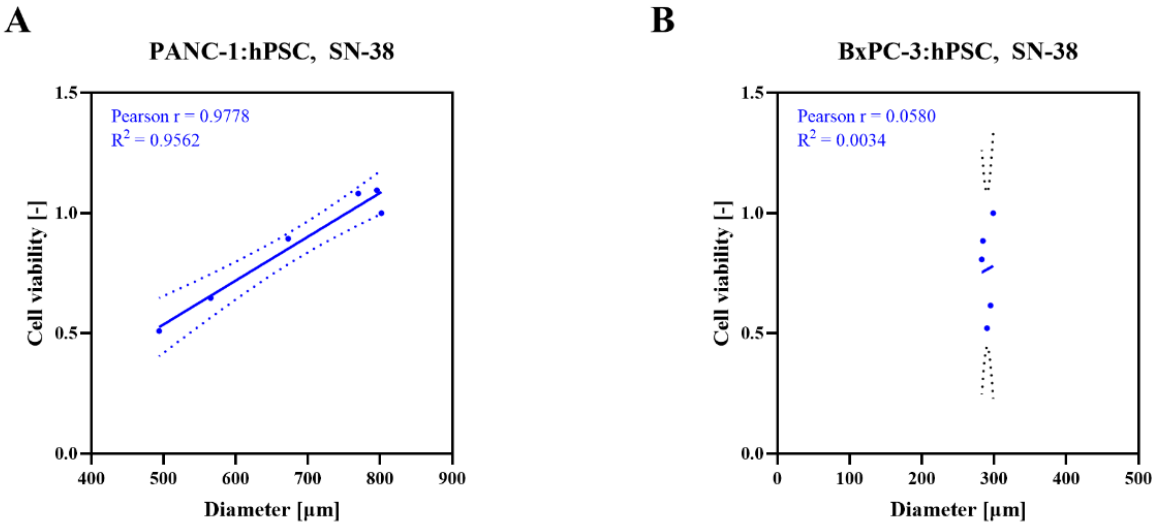

Figure S14. *Correlation between* ***A)*** *PANC-1:hPSC and* ***B)*** *BxPC-3:hPSC spheroid viability quantified by CellTiter Glo® and spheroid diameter calculated from Incucyte® images. Pearson r coefficient and* R^2 *are shown on each plot. Dotted lines represent 95% confidence intervals.*

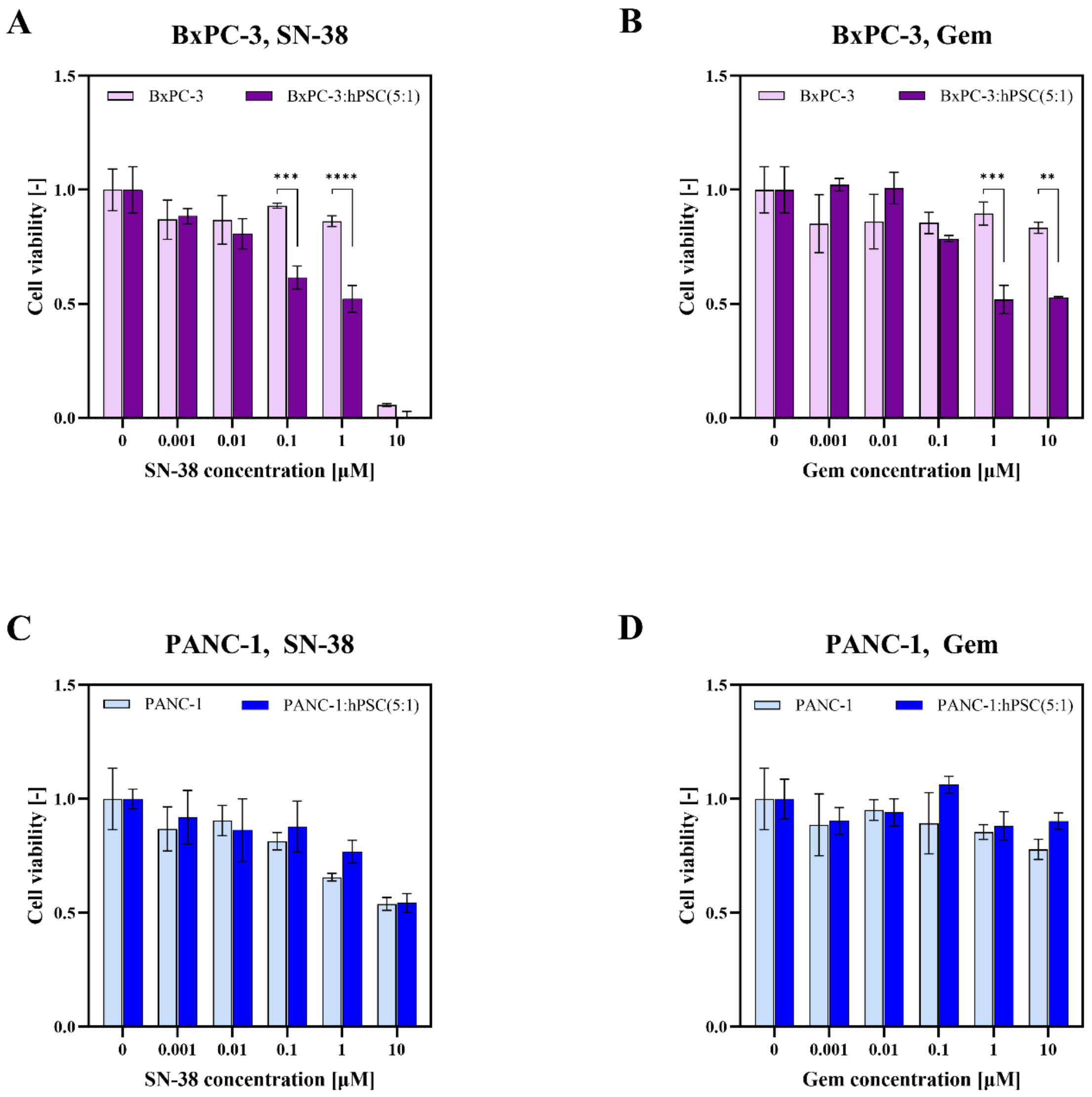

Figure S15. A) *Dose responses of BxPC-3 monoculture and BxPC-3:hPSC(5:1) co-culture spheroids to SN-38 and* ***B)*** *gemcitabine.* ***C)*** *Dose responses of PANC-1 monoculture and PANC-1:hPSC(5:1) co-culture spheroids to SN-38 and* ***D)*** *gemcitabine. Viability was quantified using CellTiter Glo® and data were compared using two-way ANOVA.*

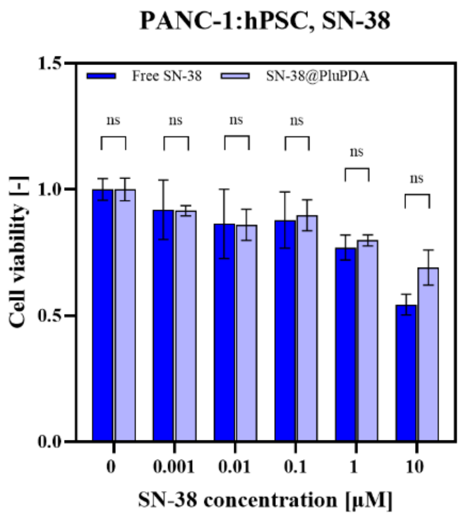

Figure S16. *Dose response of PANC-1:hPSC spheroids to free SN-38 and SN-38 encapsulated in PluPDA NCs (SN-38@PluPDA) quantified by CellTiter Glo®. Data were compared using two-way ANOVA.*

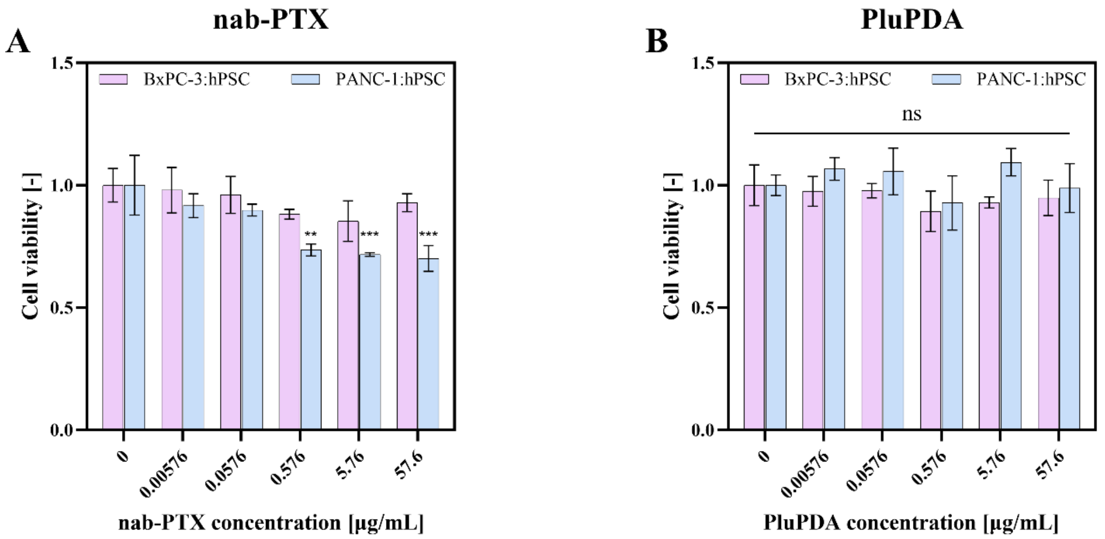

Figure S17. *Dose response of BxPC-3:hPSC and PANC-1:hPSC spheroids to* ***A)*** *nab-paclitaxel (nab-PTX) and* ***B)*** *drug-free PluPDA NCs at NC concentrations equivalent to those used in SN-38@PluPDA studies. Viability was quantified by CellTiter Glo® and data were compared using two-way ANOVA.*

Supplementary Methods

Materials list:

Material	Catalogue number	Supplier
4',6-Diamidino-2-phenylindole (DAPI)	10116287	Thermo Fisher Scientific
Dulbecco's Modified Eagle Medium (DMEM)	11995065	
Ethanol	10437341	
7-Ethyl-10-hydroxycamptothecin (SN-38)	15402262	
Gemcitabine hydrochloride	15780899	
Glutaraldehyde	11428743	
Goat anti-rabbit IgG secondary antibody (Alexa Fluor™ Plus 555)	15636746	
Goat anti-rat IgG secondary antibody (Alexa Fluor™ Plus 647)	17129078	
Corning® Matrigel® Matrix	11543550	
2-(*N*-Morpholino)ethanesulfonic acid (MES)	10419123	
Penicillin-streptomycin (5,000 U/mL)	15070063	
1X phosphate-buffered saline (PBS)	10010023	
ProLong™ Diamond Antifade Mountant	15372192	
Roswell Park Memorial Institute (RPMI) 1640 medium	11875093	
Sodium sulfate (anhydrous)	10032590	
Sucrose	10172540	
SYTOX™ Green Nucleic Acid Stain	10768273	
Toluene	10346390	
ToxiLight® Non-destructive Cytotoxicity Bioassay Kit	11640261	
Trifluoroacetic acid (TFA)	10194920	
TrypLE Express	12604013	
Ultra-low attachment 96-well plates	10023683	
Urea	10102790	
White 96-well plates	10167481	
Agarose	A9539	Merck
Bovine serum albumin (BSA)	A3059	
Dichloromethane (DCM)	32222	
Dimethylformamide (DMF)	227056	
Di-*tert*-butyl dicarbonate	361941	
Dopamine hydrochloride	H8502	
2,2′-(ethylenedioxy)bis(ethylamine)	385506	
Fetal bovine serum (FBS)	F7524	

Material	Catalogue number	Supplier
Glycerol	G7893	Merck
Paraformaldehyde (PFA)	1.00496.8350	
Rhodamine B isothiocyanate	283924	
Syringe filters (0.22 µm pore size, PES membrane)	SLGPR33RS	
Triethylamine	90335	
Tris hydrochloride	648313	
Triton X-100	T8787	
Trizma® base	T1503	
Vivaspin® 20 centrifugal concentrators (PES membrane, 30 kDa MWCO)	28-9323-61	
Methanol	20846.326	VWR
Optimal cutting temperature (OCT) compound	361603E	
Sodium carbonate (Na_2CO_3)	27771.233	
Fibronectin rabbit anti-human antibody	ab2413	Abcam
Ki-67 rabbit anti-human antibody	orb632863	Biorbyt
Nab-paclitaxel	HY-P99974	Cambridge Bioscience
Sodium bicarbonate ($NaHCO_3$)	F044705	Fluorochem
Hypoxyprobe™ Kit - Rat MAb	HP1-100Kit	Hypoxyprobe
Collagen (type I, from rat tail)	50201	Ibidi
Dialysis tubing (12-14 kDa MWCO)	DTV12000.08.30	Medicell Membranes
Dimethyl sulfoxide (DMSO)	194819	MP Biomedicals
CellTiter-Glo® Luminescent Cell Viability Assay	G7570	Promega
CoraLite® Plus 488 TUNEL Assay Apoptosis Detection Kit (green)	PF00006	Proteintech

Nanocarrier synthesis and characterization: PluPDA NCs were synthesized using a co-polymerization method previously developed in our lab[1]. Briefly, Trizma® base (22.5 mg) was dissolved in 2.5 mL of water, mixed with 10.5 mL ethanol and 19.5 mL of water, then stirred at room temperature for 30 min. Separately, dopamine hydrochloride (13.7 mg) and a dopamine-Pluronic® F127 conjugate (45.7 mg) (prepared as described in [44]) were dissolved in 1 mL of ethanol each, mixed, sonicated, and added dropwise to the Trizma solution. The reaction mixture was then left stirring at room temperature overnight, resulting in a dark brown NC suspension. Finally, the NCs were washed with water using Vivaspin® 20 centrifugal concentrators (PES membrane, 30 kDa MWCO) until the filtrate was colorless (2,900 RCF, 30

min per wash). NC hydrodynamic size was confirmed using a BeNano 180 Zeta Pro (Bettersize) dynamic light scattering instrument. For storage, the NCs were snap-frozen in liquid nitrogen and lyophilized using a VirTis AdVantage Plus benchtop freeze dryer (ATS Scientific Products).

Nanocarrier labelling with rhodamine B: PluPDA NCs were labelled with rhodamine B using a Michael addition-based method previously developed in our lab[44]. Briefly, lyophilized PluPDA NCs (5 mg) were re-dispersed in 5 mL of tris buffer (10 mM). Separately, rhodamine B-TEG-NH_2 (10 mg) (synthesis described in **Supplementary Materials & Methods**) was dissolved in 0.5 mL of ethanol and added dropwise to the NC suspension. The mixture was stirred overnight at room temperature in the dark, then washed with water using Vivaspin® 20 centrifugal concentrators (PES membrane, 30 kDa MWCO) until the filtrate was colorless (2,900 RCF, 30 min per wash) and dialyzed against water for 72 h using 12-14 kDa MWCO dialysis tubing. Rhodamine attachment was confirmed via fluorescence spectroscopy using a CLARIOstarPlus plate reader (BMG Labtech).

Nanocarrier loading with SN-38: 4 mL of PluPDA NCs suspended in water (6 mg/mL) were mixed with 4 mL of $NaHCO_3/Na_2CO_3$ buffer (100 mM, pH 9.5). Separately, SN-38 (14.8 mg) was dissolved in 0.5 mL DMSO and added dropwise to the NPs, and the mixture was left stirring at room temperature overnight. The mixture was then centrifuged at high speed to precipitate the non-dissolved excess SN-38, which was discarded. The NC-containing supernatant was then washed twice with MES buffer (5 mM, pH 4.5) and twice with water using Vivaspin® 20 centrifugal concentrators (PES membrane, 30 kDa MWCO) (2,900 RCF, 30 min per wash). The washed NC suspension was then passed through a syringe filter (0.22 μm pore size, PES membrane) and its SN-38 content was determined via UV-Vis using a CLARIOstarPlus plate reader (BMG Labtech).

Synthesis of rhodamine B-TEG-NH_2: The rhodamine B-TEG-NH_2 used to label PluPDA NCs was synthesized in-house via the three-step process summarized below. All NMR measurements were performed using a 400 MHz QNP Cryoprobe Spectrometer (Bruker) by the NMR service of the Yusuf Hamied Department of Chemistry, University of Cambridge. Mass spectrometry analyses were

performed by the MS service of the Yusuf Hamied Department of Chemistry, University of Cambridge using a range of Agilent and Waters instruments.

Synthesis of Compound (1)

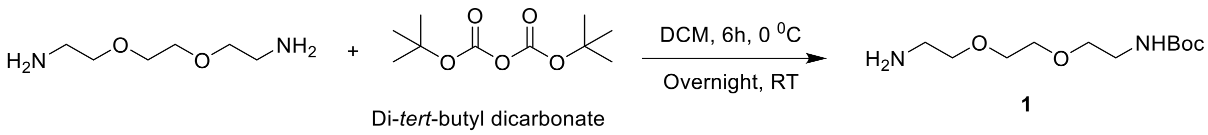

Compound **1** was synthesized according to a reported method with slight modification[2]. A solution of di-*tert*-butyl dicarbonate (11.0 g, 60.0 mmol) in 250 mL CH_2Cl_2 was added dropwise to a solution of 2,2′-(ethylenedioxy)bis(ethylamine) (30.0 mL, 200 mmol) in 200 mL dry CH_2Cl_2 at 0 °C under nitrogen atmosphere over a period of 6 h. The reaction mixture was stirred at 0°C for 6 h and then at room temperature overnight. The mixture was extracted with 200 mL brine three times and 200 mL water. The organic phase was collected and dried over Na_2SO_4. The solvent was evaporated under vacuum to give a colourless oil (6.1 g, 71%).

^{1}H NMR (400 MHz, $CDCl_3$): δ (ppm) 1.41 (s, 9H). 2.61 (t, *J* = 5.3 Hz, 2H), 3.02 (m, 2H), 3.24-3.36 (m, 4H), 3.38-3.44 (m, 4H), 5.41 (br, 1H).

HR-MS (ESI) for $C_{11}H_{24}N_2O_4[M]^+$: 248.1728.

Synthesis of Compound (2)

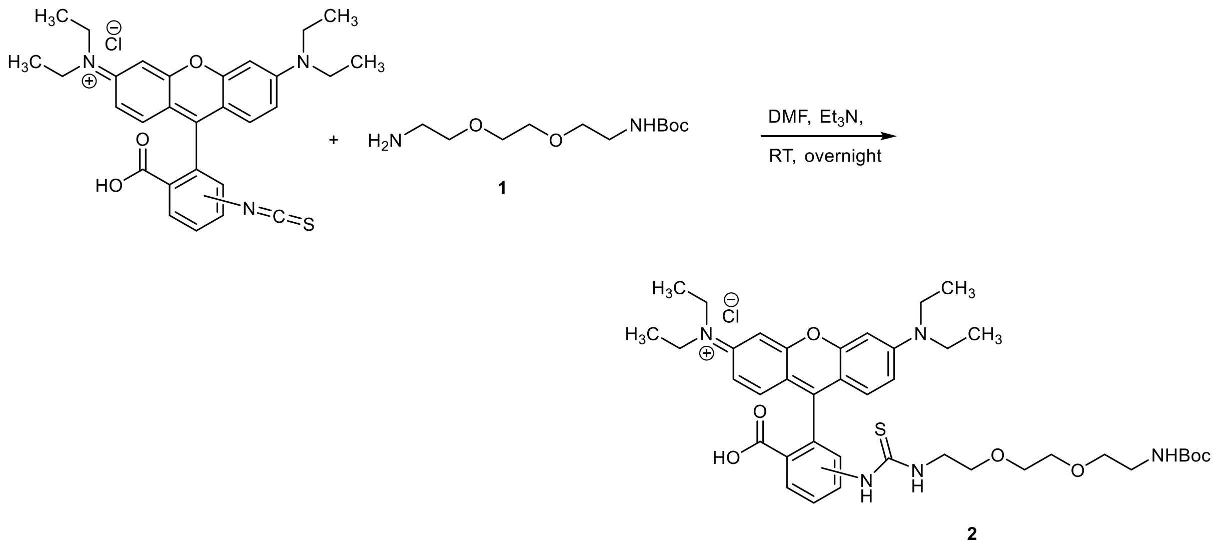

Compound **1** (200 mg, 0.806 mmol) was dissolved in anhydrous DMF (10 mL) in an oven dried 2-neck flask under argon and triethylamine (244 mg, 328 µL, 2.410 mmol) was added. The solution was cooled in an ice bath and a solution of rhodamine B isothiocyanate (mixed isomers) (216 mg, 0.403 mmol) in DMF (10 mL) was added to the solution dropwise over a period of 30 min. After complete

addition, the ice bath was removed and the reaction mixture was stirred overnight at room temperature. The solvent was removed under vacuum to give a dark purple residue. Silica gel column chromatography using CH_2Cl_2:MeOH (9:1) gave pure protected compound **2** as a thick purple oil (280 mg, 0.374 mmol, 92 % yield).

^{1}H NMR (400 MHz, $CDCl_3$, mixture of isomers): δ (ppm) 1.16 (t, *J* = 7.2 Hz, 6H), 1.26 (t, *J* = 7.2 Hz, 6H), 1.41 (s, 9H), 1.81 (m, 4H), 3.11 (br, 2H), 3.22-3.38 (m, 8H), 3.49-378 (m, 12H), 6.29 (d, *J* = 1.7 Hz, 1H), 6.37 (dd, *J* = 2.4 Hz, 8.8 Hz, 2H), 6.43 (d, *J* = 2.4 Hz, 1H), 6.66 (br, 2H), 6.71-7-75 (m, 3H), 6.83 (d, *J* = 9.3 Hz 2H), 6.98 (d, *J* = 8.3 Hz, 1H), 7.33 (d, *J* = 9.3 Hz, 2H), 7.70 (d, *J* = 8.3 Hz, 1H), 7.82 (br, 1H), 8.25 (br, 1H), 8.83 (br, 2H), 10.84 (bs, 2H).

^{13}C NMR (100 MHz, $CDCl_3$, mixture of isomers): δ (ppm) 12.6, 13.7, 25.6, 28.4, 39.6, 40.2, 44.6, 45.7, 66.9, 67.9, 70.0, 70.2, 70.3, 79.2, 95.7, 95.8, 97.2, 107.5, 107.7, 108.1, 108.6, 113.2, 113.9, 115.9, 117.9, 122.7, 126.9, 129.2, 129.7, 149.1, 149.3, 152.7, 153.9, 154.3, 156.4, 157.9, 162.7, 180.9.

HR-MS (ESI) for $C_{40}H_{55}N_5O_5S$ $[M+H]^+$: 749.5245.

Synthesis of Rhodamine B-TEG-NH₂

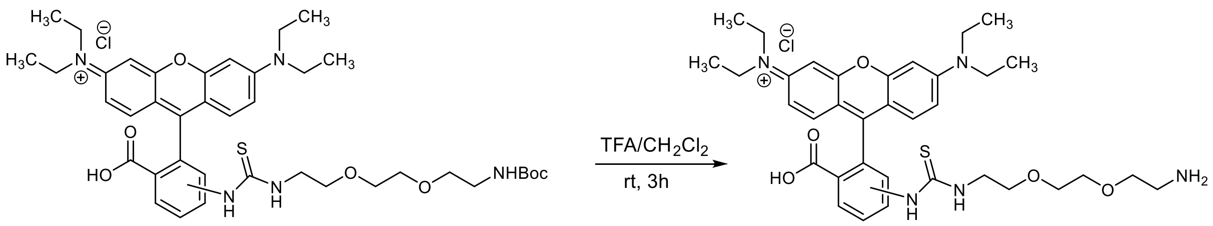

2 **Rh-TEG-NH$_2$ (3)**

To a solution of Boc-protected compound **2** (200 mg, 0.267 mmol) in dichloromethane (20 mL) was added trifluoroacetic acid (6 mL). The mixture was stirred for 3 h at room temperature. The solvent was evaporated under reduced pressure to give a yellow residue. Dichloromethane (20 mL) was added to the residue and evaporated. This process was repeated three times (3x20 mL) to remove the trifluoroacetic acid. Toluene (30 mL) was added to the residue and the solvent was evaporated to remove any traces of trifluoroacetic acid, giving a thick purple oil of Rh-TEG-NH_2 as its trifluoroacetate salt (170 mg, 0.264 mmol, 96 % yield).

^{1}H NMR (400 MHz, $CDCl_3$, mixture of isomers): δ (ppm) 1.15 (t, *J* = 7.1 Hz, 6H), 1.27 (t, *J* = 7.1 Hz, 6H), 1.80 (m, 4H)3.09 (br, 2H), 3.26-3.37 (m, 8H), 3.45-373 (m, 28H), 6.28 (d, *J* = 1.7 Hz, 1H), 6.38 (dd, *J* = 2.4 Hz, 8.8 Hz, 2H), 6.41 (d, *J* = 2.4 Hz, 1H), 6.65 (br, 2H), 6.70-7-75 (m, 3H), 6.83 (d, *J* = 9.3 Hz, 2H), 6.96 (d, *J* = 8.3 Hz, 1H), 7.31 (d, *J* = 9.3 Hz, 2H), 7.71 (d, *J* = 8.3 Hz, 1H), 7.80 (br, 1H), 8.22 (br, 1H), 8.81 (br, 2H), 10.81 (bs, 2H).

^{13}**C NMR** (100 MHz, $CDCl_3$, mixture of isomers): δ (ppm) 12.5, 12.6, 39.6, 40.2, 44.6, 45.7, 66.9, 67.9, 70.0, 70.2, 70.3, 95.7, 95.8, 97.2, 107.5, 107.7, 108.1, 108.6, 113.2, 113.9, 115.9, 117.8, 121.9, 126.9, 129.2, 129.4, 149.5, 149.9, 152.3, 153.6, 154.8, 156.1, 157.8, 180.2.

HR-MS (ESI) for $C_{35}H_{47}N_5O_5S$ $[M+H]^+$: 649.21439.

References:

[1] A. B. Popov, F. Melle, E. Linnane, C. González-López, I. Ahmed, B. Parshad, C. O. Franck, H. Rahmoune, F. M. Richards, D. Muñoz-Espín, D. I. Jodrell, D. Fairen-Jimenez, L. Fruk, *Nanoscale* **2022**, *14*, 6656.

[2] Z. Zeng, S. Mizukami, K. Kikuchi, *Anal Chem* **2012**, *84*, 9089.

Supplementary movie files

Supplementary Video 1. *Time-lapse Incucyte imaging of the growth of a PANC-1:hPSC spheroid in the absence of Matrigel.*

Supplementary Video 2. *Time-lapse Incucyte imaging of the growth of a PANC-1:hPSC spheroid supplemented with 2.5% Matrigel.*

Supplementary Video 3. *Time-lapse Incucyte imaging of the growth of a BxPC-3:hPSC spheroid in the absence of Matrigel.*

Supplementary Video 4. *Time-lapse Incucyte imaging of the growth of a BxPC-3:hPSC spheroid supplemented with 2.5% Matrigel.*

Supplementary Video 5. *Composite z-stack confocal laser scanning microscopy video of a BxPC-3:hPSC spheroid incubated with rhodamine B-labeled polydopamine-Pluronic F127 NCs and stained with the nuclear dye SYTOX™ Green. The rhodamine B channel is shown up to a depth of 150 µm and the scale bar represents 100 µm.*

Supplementary Video 6. *Composite z-stack confocal laser scanning microscopy video of a BxPC-3:hPSC spheroid incubated with rhodamine B-labeled polydopamine-Pluronic F127 NCs and stained with the nuclear dye SYTOX™ Green. The SYTOX™ Green channel is shown up to a depth of 150 µm and the scale bar represents 100 µm.*

Supplementary Video 7. *Composite light sheet fluorescence microscopy video of a BxPC-3:hPSC spheroid incubated with rhodamine B-labeled polydopamine-Pluronic F127 NCs and stained with the nuclear dye SYTOX™ Green. The rhodamine B channel is shown up to a depth of 400 µm and the scale bar represents 100 µm.*

Supplementary Video 8. *Composite light sheet fluorescence microscopy video of a BxPC-3:hPSC spheroid incubated with rhodamine B-labeled polydopamine-Pluronic F127 NCs and stained with the nuclear dye SYTOX™ Green. The SYTOX™ Green channel is shown up to a depth of 400 µm and the scale bar represents 100 µm.*

Supplementary Video 9. *Composite z-stack confocal laser scanning microscopy video of a PANC-1:hPSC spheroid incubated with rhodamine B-labeled polydopamine-Pluronic F127 NCs and stained with the nuclear dye SYTOX™ Green. The rhodamine B channel is shown up to a depth of 150 µm and the scale bar represents 100 µm.*

Supplementary Video 10. *Composite z-stack confocal laser scanning microscopy video of a PANC-1:hPSC spheroid incubated with rhodamine B-labeled polydopamine-Pluronic F127 NCs and stained with the nuclear*

dye SYTOX™ Green. The SYTOX™ Green channel is shown up to a depth of 150 µm and the scale bar represents 100 µm.

Supplementary Video 11. *Composite light sheet fluorescence microscopy video of a PANC-1:hPSC spheroid incubated with rhodamine B-labeled polydopamine-Pluronic F127 NCs and stained with the nuclear dye SYTOX™ Green. The rhodamine B channel is shown up to a depth of 500 µm and the scale bar represents 100 µm.*

Supplementary Video 12. *Composite light sheet fluorescence microscopy video of a PANC-1:hPSC spheroid incubated with rhodamine B-labeled polydopamine-Pluronic F127 NCs and stained with the nuclear dye SYTOX™ Green. The SYTOX™ Green channel is shown up to a depth of 500 µm and the scale bar represents 100 µm.*

Supplementary Video 13: *Time-lapse Incucyte imaging of the growth of a A549:WI-38 spheroid in the absence of Matrigel.*

Supplementary Video 14: *Time-lapse Incucyte imaging of the growth of a A549:WI-38 spheroid supplemented with 2.5% Matrigel.*
